# Species Delimitation and Conservation in Taxonomically Challenging Lineages: The Case of Two Clades of *Capurodendron* (Sapotaceae) in Madagascar

**DOI:** 10.3390/plants10081702

**Published:** 2021-08-18

**Authors:** Carlos G. Boluda, Camille Christe, Aina Randriarisoa, Laurent Gautier, Yamama Naciri

**Affiliations:** 1 Conservatoire et Jardin botaniques de la Ville de Genève, Chemin de l’Impératrice 1, Chambésy, 1292 Geneva, Switzerland; camille.christe@ville-ge.ch (C.C.); aina.randriarisoa@ville-ge.ch (A.R.); laurent.gautier@ville-ge.ch (L.G.); yamama.naciri@ville-ge.ch (Y.N.); 2 Laboratoire de Systématique végétale et Biodiversité, Université de Genève, Chemin de l’Impératrice 1, Chambésy, 1292 Geneva, Switzerland

**Keywords:** conservation, current speciation, hybridization, species complex, species delimitation

## Abstract

*Capurodendron* is the largest endemic genus of plants from Madagascar, with around 76% of its species threatened by deforestation and illegal logging. However, some species are not well circumscribed and many of them remain undescribed, impeding a confident evaluation of their conservation status. Here we focus on taxa delimitation and conservation of two species complexes within *Capurodendron*: the Arid and Western complexes, each containing undescribed morphologies as well as intermediate specimens alongside well-delimited taxa. To solve these taxonomic issues, we studied 381 specimens morphologically and selected 85 of them to obtain intergenic, intronic, and exonic protein-coding sequences of 794 nuclear genes and 227 microsatellite loci. These data were used to test species limits and putative hybrid patterns using different approaches such as phylogenies, PCA, structure analyses, heterozygosity level, FST, and ABBA-BABA tests. The potential distributions were furthermore estimated for each inferred species. The results show that the *Capurodendron* Western Complex contains three well-delimited species, *C. oblongifolium*, *C. perrieri*, and *C. pervillei*, the first two hybridizing sporadically with the last and producing morphologies similar to, but genetically distinct from *C. pervillei*. The Arid Complex shows a more intricate situation, as it contains three species morphologically well-delimited but genetically intermixed. *Capurodendron mikeorum* nom. prov. is shown to be an undescribed species with a restricted distribution, while *C. androyense* and *C. mandrarense* have wider and mostly sympatric distributions. Each of the latter two species contains two major genetic pools, one showing interspecific admixture in areas where both taxa coexist, and the other being less admixed and comprising allopatric populations having fewer contacts with the other species. Only two specimens out of 172 showed clear genetic and morphological signals of recent hybridization, while all the others were morphologically well-delimited, independent of their degree of genetic admixture. Hybridization between *Capurodendron androyense* and *C. microphyllum*, the sister species of the Arid Complex, was additionally detected in areas where both species coexist, producing intermediate morphologies. Among the two complexes, species are well-defined morphologically with the exception of seven specimens (1.8%) displaying intermediate patterns and genetic signals compatible with a F1 hybridization. A provisional conservation assessment for each species is provided.

## 1. Introduction

Species conservation assessments, as currently conducted on a wide scale using the International Union for Conservation of Nature (IUCN) criteria, are based on distribution data of clearly defined species and have proved to be a useful pragmatic tool. However, the species concept is sometimes subjective, especially when there is a mismatch between morphospecies (understood as a morphologically delimited group, described or not, considered potentially a valid species and meriting further evaluation) and genetic lineages. Depending on which concept is used, the number of final units to be conserved can vary greatly. With massive DNA sequencing, we can now use unprecedented amounts of genetic information; however, how much this information can help us to establish clear and practical species limits in critical cases is still an open question.

With 33 described species so far and more than a dozen to come, *Capurodendron* (Sapotaceae) is the largest endemic genus of plants in Madagascar [1,2]. It contains trees, rarely shrubs, growing from the most humid to the driest areas of the island, with a great variety of leaf morphology but a highly conserved flower architecture [3]. *Capurodendron*, like most other Sapotaceae, usually produces a reddish hard wood resistant to insect and microorganism damage, and it is therefore highly appreciated locally for furniture and carpentry [4,5]. At the international level, numerous American, Asian and Continental African Sapotaceae species are traded and highly valued. In Madagascar, although trade had essentially developed at the local and national scales so far, signs of illegal logging for overseas exportation have been detected (R. Randrianaivo, pers. comm.). Together with ebonies (*Diospyros* spp.), exportation is thus expected to increase as other precious timbers such as rosewood (*Dalbergia* spp.) become scarcer [6]. Selective logging, together with the massive ongoing deforestation of Madagascar [7,8] has led to 76% of the *Capurodendron* species being threatened according to the IUCN criteria, with even one species out of four being Critically Endangered or possibly Extinct [1,9].

The lack of a robust taxonomy has affected the conservation assessment of many *Capurodendron* species, as for example *C. ludiifolium*, which was considered only as Vulnerable (VU) a few years ago [10]. However, after the revision of Boluda et al. [1], *Capurodendron ludiifolium* was split into five unrelated species (*C. ludiifolium*, *C. naciriae*, *C. sahafariense*, *C. randrianaivoi*, and *C. sakarivorum*), illustrating a case of evolutionary convergence toward a similar leaf venation. Of these five taxa, three are now considered Endangered (EN) and two Critically Endangered (CR). Other examples include two recently described local endemic species [1], both assessed as CR, and which were previously confounded with widespread and genetically distant species: *Capurodendron andrafiamenae* with *C. greveanum* (Least Concern, LC) [11] and *C. birkinshawii* with *C. nodosum*, (Vulnerable, VU) [12]. The latter case additionally highlights the tremendous impact of incorrect taxonomy on extent of occurrence (EOO) calculations. *Capurodendron nodosum* is indeed restricted to the extreme north of Madagascar, while the only known specimen of *C. birkinshawii* was collected in the extreme south. Including the latter in the EOO calculation of the former would have therefore erroneously raised the EOO value from ca. 3000 to 58,000 km^2^.

While a 638 gene-based phylogeny showed clear species limits across the major part of the genus *Capurodendron* [1], three species complexes still remain unresolved, impeding the conservation assessment of the taxa they contain. One of them has been named the Eastern Complex as it is found all along the eastern moist evergreen forests of Madagascar. It comprises the morphologically variable species *Capurodendron tampinense*, which according to genetic data, seems to constitute a group of morphologically similar but genetically different species [1]. The resolution of this complex will however require further sampling. In this paper we focus on the resolution of the two other groups, the Western Complex and the Arid Species Complex.

The Western Complex (Table 1) occurs in the deciduous forests of western Madagascar below 300 m elevation and contains three genetically related species, *Capurodendron oblongifolium*, *C. perrieri* and *C. pervillei*, and a fourth undescribed morphospecies similar to *C. pervillei* and here referred to as *C. aff. pervillei*. *Capurodendron oblongifolium* was originally described as a variety of *C. perrieri* [13], then subsumed in the *Flore de Madagascar* Sapotaceae treatment [3]. It has been recently resurrected as a distinct species [1]. Although *Capurodendron oblongifolium* and *C. perrieri* grow in similar habitats, they are allopatric: *C. perrieri* is more widespread and with rare exception is found <50 km from the coast in the regions of Menabe, Melaky and Boeny, while *C. oblongifolium* is always >100 km inland and is restricted to Boeny. *Capurodendron pervillei* grows sympatrically with the two above-mentioned species but is morphologically well differentiated. Finally, the undescribed morphospecies is only known from two specimens embedded in the *Capurodendron pervillei* distribution area, one specimen found among populations of *C. oblongifolium* and the other among populations of *C. perrieri*. Previous phylogenies [14] found the undescribed morphospecies to be polyphyletic, suggesting that more than one taxon may present this morphology.

The Arid Complex (Table 1) mainly contains two morphologically well-differentiated species: *Capurodendron androyense* is restricted to the southern and southwestern sub-arid ecosystems, while *C. mandrarense* also extends inland to seasonally dry habitats up to 1000 m altitude. An additional morphospecies occurs in the northwestern edge of the subarid zone which is phenotypically intermediate between, and alternatively identified as, *Capurodendron mandrarense* and *C. greveanum*, the latter being a distantly related widespread species along the western and northern coast. This morphospecies is hereafter called *Capurodendron greveanum-mandrarense*.

The specimens of this Arid Complex form a monophyletic clade sister to *Capurodendron microphyllum*, which has a restricted distribution in sympatry at the extreme southeast of the Arid Complex area. This species is morphologically different from the former ones, although a few specimens exhibit morphologies related to *Capurodendron androyense*, suggesting that hybridization could sporadically occur. The Arid Complex presents two main taxonomical problems: First, the morphospecies *Capurodendron androyense*, *C. mandrarense* and *C. greveanum-mandrarense* might be considered conspecific as they appear intermixed in previously reconstructed phylogenies, showing a mismatch between morphology and detected genetic lineages [1,14]. Second, *Capurodendron greveanum* is a species phylogenetically and morphologically clearly distinct from *C. mandrarense*, and consequently *C. greveanum-mandrarense* is unlikely to represent intermediate morphologies uniting both taxa as a single species. The morphology of *Capurodendron greveanum-mandrarense* could be the result of hybridization events; however, it is absent from the area were the putative parental species coexist.

The goal of this paper is to delimit the taxa of the *Capurodendron* Western and Arid species complexes and explore how the species concept can be applied to lineages in which species are incompletely isolated. For this, we extend the previous use of exonic genetic markers by Christe et al. [14] to intronic and intergenic ones, as well as to microsatellites (STR), all showing much higher substitution rates than exons alone. We aim to improve the resolution at the population level and address genetic admixture, introgression and hybridization in order to discuss how the IUCN criteria for species conservation ([15] IUCN Species Survival Commission, 2012) can be implemented to ensure the preservation of the genetic diversity of species complexes.

## 2. Materials and Methods

Taxon sampling—*Capurodendron* herbarium samples stored in G, K, MO, P, TAN and TEF herbaria (ca. 860 gatherings) were morphologically studied and specimens that did not belong to the two target species complexes were discarded, retaining 381 specimens, 43 of which being collected in 2017 during a dedicated field trip in southern Madagascar. Dry specimens were morphologically analyzed using a stereomicroscope (max. 65x), and characteristics of fresh material annotated in the field or deduced from accompanying pictures, when available. Flowers and fruits were boiled 2–10 min to rehydrate them and restore their three-dimensional shape or to isolate the seed. Out of the 381 specimens, 85 (52% from silica-gel preserved specimens and 48% from old herbarium material) representing all the morphological, ecological and geographical variability within species, were selected for DNA extraction: 15 belonged to the Western Complex (*Capurodendron oblongifolium*, *C. perrieri*, *C. pervillei*, and *C. aff. pervillei*) and 57 to the more intricate Arid Complex (*C. androyense*, *C. greveanum*, *C. greveanum-mandrarense*, *C. mandrarense*, *C. microphyllum*). Thirteen specimens belonging to the closest species of both complexes (*Capurodendron gracilifolium*, *C. nanophyllum*, *C. rubrocostatum* and *C.* sp. 20) and three outgroups (*C. birkinshawii*, *C. delphinense* and *Bemangidia lowry*) were added for the phylogenetic study (Table 2).

Ordination of Morphological data—In order to objectify identifications in the intricate Arid Complex, morphological data were gathered on an expanded number of specimens. A total of 22 characters (Table 3) were scored across 123 specimens (Table S1). For quantitative characters that displayed variability within a single specimen (e.g., leaf length or number of secondary nerves) an average value of 10 measures was used, while for qualitative variable characters (e.g., type of leaf apex) we selected the dominant state on the specimen. To allow data ordination using qualitative and quantitative variables at once, Factorial Analysis for Mixed Data (FAMD) was conducted and run using the R package FactoMineR [16]; http://factominer.free.fr, accessed on 12 August 2021). This approach was considered unnecessary for the Western Complex.

DNA sequencing—DNA was extracted using the CTAB method with chloroform, including sorbitol washes to remove mucilaginous substances [14,17,18]. The sequences were obtained following the methodology explained in Christe et al. [14] combining gene capture with Next Generation Sequencing. For this, a genomic library of each specimen was constructed and labelled with dual indexing barcodes. Specimens were then pooled and 794 protein coding genes and 227 microsatellite loci were captured using a hybridization step with specific biotinylated oligonucleotide probes complementary to the loci of interest. Hybridized sequences were retained by streptavidin-covered magnetic beads while all non-target DNA was washed away. Finally, captured DNA was sequenced using an Illumina HiSeq 4000 machine (2 × 100 bp paired-end).

Capture data processing—The quality of DNA reads was checked with FASTQC (https://www.bioinformatics.babraham.ac.uk/projects/fastqc/, accessed on 12 August 2021) and they were trimmed with Trimmomatic version 0.38 [19]. In order to explore our study question with different type of markers displaying different substitution rates, we extracted our targeted exonic loci as well as the flanking intronic regions when present, as the latter have a higher mutation rate than exons [20]. We also extracted the sequences around the STR loci, which consisted of intergenic non-coding DNA. Four different datasets were gathered: (1) exons, (2) supercontigs (exonic and intronic sequences), (3) STR flanking regions, and (4) STR loci. The aligned sequences and SNPs (single nucleotide polymorphisms) were extracted for the three first datasets.

*Aligned sequences*—The program HybPiper [21] was run to obtain the 792 nuclear loci and 227 sequences containing STR loci presented in Christe et al. [14], in order to extract the consensus sequence of these loci for all individuals. For the 792 nuclear loci, the same program was run with the intronerate.py script [21] in order to get the supercontig sequences. All these sequences were aligned using the program MAFFT version 7 [22]. Putative paralogs according to Hybpiper were remove, resulting in 638 aligned nuclear loci.

*SNPs—*For 792 nuclear loci, the longest consensus sequence of each gene was selected as a reference for mapping the reads of each individual in order to extract the SNPs. BWA version 0.7.16 [23] was used for mapping, followed by Picard version 2.21.1 and Samtools version 1.9 [24,25] to sort, remove duplicates, and to index. SNPs and indels were called separately for each individual with HaplotypeCaller from GATK version 4.1.3. The resulting gvcf files were combined and genotyped with the same program. The resulting vcf files were filtered with vcftools version 0.1.16 [26], after removal of putative paralog loci, with the following settings: --minDP 8 --remove-indels --min-alleles 2 --max-alleles 2 --max-missing 0.8). For STR flanking regions, the same strategy was used to extract the SNP, with additionally removing the STR regions with vcftools using a bed file of the concerned positions.

*Microsatellites—*STR dataset was extracted according to Highnam et al. [27]. Trimmed reads were first mapped to the reference sequence of the STR (STR + flanking region) with Bowtie2 version 2.3.4.2 [28] followed by Picard version 2.21.1 and Samtools version 1.9 to sort, remove duplicates, and to index. Genotyping was accessed with RepeatSeq [27].

In order to exclude polyploidy in some problematic samples, we used the program nQuire [29] to estimate ploidy in each specimen. This method has been used successfully in target capture data for herbarium samples [30].

Phasing—To be able to reconstruct phylogenies using both alleles for each specimen (instead of a consensus sequence) we performed a phasing analysis. For that, supercontigs (containing exons and flanking intronic sequences) for each gene and specimen were obtained using the reads_first.py and intronerate.py scripts of the HybPiper pipeline [14,21]. Then these supercontigs were used as reference sequences for identifying variants for each specimen according to Kates et al. ([31] 2018, pipeline available at https://github.com/mossmatters/phyloscripts/tree/master/alleles_workflow, accessed on 12 August 2021). To assemble the alleles, WhatsHap [32], a Python-based program, combined with Tabix 0.2.6 (https://sourceforge.net/projects/samtools/files/tabix/, accessed on 12 August 2021) were run, and phased sequences were then converted into fasta files using bcftools consensus [33]. As a complete phasing was not expected, especially when using short DNA fragments as here, we retained the biggest phased block of each gene and replaced the remaining sequence by the consensus using haplonerate.py (Kates et al., 2018 [31]; https://github.com/mossmatters/phyloscripts, accessed on 12 August 2021). At the end we obtained two partially or completely phased sequences per gene for each specimen, except for the homozygous loci.

As there is no way to know which allele at a given locus is linked to any other allele at another locus, only gene trees can be estimated, and not species tree. Gene trees of the 638 loci without paralogy signals [13] were performed using RAXML v.8.2.4 [34] with a GTRGAMMA substitution model, discarding nucleotide positions with more than 20% missing data. All the generated trees were manually examined searching for the topological location of each allele for the specimens of interest (e.g., *Capurodendron* aff. *pervillei*).

Heterozygosity—In order to detect individuals with special features such as polyploidy or recent hybridization, we measured the heterozygosity level of each specimen with vcftools version 0.1.16 [26] on each SNP dataset. We calculated the percentage of observed heterozygosity as follows: (total number of sites - homozygotic sites observed)/total number of sites.

Phylogenetic reconstructions.—Out of the 85 specimens, those with more than 20% of loci missing were removed and, for the specimens that were retained, positions missing more than 20% were similarly removed. Phylogenetic reconstructions were performed using three different datasets: A) 600 exonic gene sequences all containing the same 81 specimens, B) 608 genes containing exonic and flanking intronic sequences from 36 to 81 specimens, and C) 195 microsatellite loci flanking regions with 76 to 81 specimens. 

A gene tree for each locus was generated using RAXML v.8.2.4 [34] with a GTRGAMMA substitution model. Then Astral-II [35,36], a method based on the multispecies coalescence (MSC), was used to infer the species tree from the gene trees.

We additionally used SplitsTree4 [37] to infer a Neighbor-net network using concatenated sequences and uncorrected P-distances. For phased loci phylogeny see the Phasing section above.

Microsatellites clustering—STRUCTURE v.2.3.4 [38,39] was run on two different datasets. The first one contained the Western Complex, with *Capurodendron oblongifolium*, *C. perrieri*, *C. pervillei* and *C.* aff. *pervillei*, and the second dataset the Arid Complex, with *C. androyense*, *C. mandrarense*, *C. greveanum-mandrarense*, the closely related species *C. microphyllum* and the genetically far but morphologically related *C. greveanum*. Only specimens with less than 18% missing microsatellites and loci with less than 10% missing data were used, leading to the use of 15 specimens and 59 loci in the Western Complex (100% and 26%, respectively), and 52 specimens and 105 loci in the Arid Complex (91% and 46%, respectively). 

STRUCTURE was run with 5 million burn-in generations and 5 million iterations, using a *k* value from 1 to 10 with 5 replicates for each *k*. Runs of each *k* value were combined with CLUMMP v.1.1.2 [40]. The ΔK method of STRUCTURE HARVESTER [41] was used to estimate which k value best adjust to our data.

Ordination of genetic data—Principal Coordinate Analyses (PCA) of genetic data were computed with the package smartPCA [42] using plink formatted merged vcf files and the same three datasets as the ones used in the phylogenetic reconstruction, selecting only specimens with less than 20% missing data. 

In order to investigate the relationships within the Arid Complex as well as potential internal gene flow, we performed additional analyses using the three morphospecies and clusters based on PCA results from exons and flanking SSR datasets. For accessing the degree of genetic polymorphism, we calculated nucleotide diversity (π), and for genetic differentiation, the weighted pairwise FST. Both analyses were calculated for each site and averaged over all sites using vcftools version 0.1.16. Allele sharing between the putative parental species of *Capurodendron greveanum-mandrarense,* (*C mandrarense* and *C. greaveanum)* as well as within the Arid Complex was accessed with the Patterson’s D statistics (ABBA-BABA test) for all possible trios with Dsuite version 0.4 [43] on the exon dataset. Two subgroups were used within *Capurodendron androyense* and *C. mandrarense*, and one in *C. greveanum-mandrarense* (see results). *Capurodendron delphinense* was used as an outgroup. The statistic test Dmin was also used to infer the lower bound of D value for each trio. A significant positive Dmin means that the sharing of derived alleles between the three taxa is inconsistent with a single species-tree relating them, even in presence of incomplete lineage sorting [43,44]. Statistical significance was accessed with the Bonferroni correction and the false discovery rate (FDR) with the Benjamini–Hochberg correction.

Potential species distribution—The potential species distribution for each taxa containing more than three specimens (the minimum required for computation) was calculated with Maxent v.3.3.3a [45]. The 19 environmental variable layers BIO1 to BIO19 from Madagascar, with a spatial resolution of 30 arcsec (about 1 km^2^), were obtained from the WordClim database [46], using the raster package in R ([47] R Core Team 2013; https://cran.r-project.org/web/packages/raster/raster.pdf, accessed on 12 August 2021). The BIL layer format was transformed to Esri.asc using DIVA-GIS [48]. Each analysis was run ten times, and the median value of all runs was plotted. Only collections with confident identification were used, with 90 collection points for *Capurodendron androyense*, 79 for *C. greveanum*, 22 for *C. greveanum*-*mandrarense,* 60 for *C. mandrarense*, 14 for *C. microphyllum*, 6 for *C. oblongifolium*, 46 for *C. perrieri*, and 37 for *C. pervillei*.

## 3. Results

DNA sequences.—From the 85 analyzed specimens, 72 (85%) provided less than 5% missing data for exon sequences, 10 (12%) between 5–40% missing data, 2 (2%: specimens 162 and 194) between 40–80% and one (1%, specimen 150) more than 80%. Missing data in intronic sequences were usually higher, as our probes were designed specifically to hybridize with exonic loci. Of the 794 protein coding genes, 156 showed putative paralogy signals in one or more specimens and were discarded, thus leaving 638 genes for further analyses.

**Figure 1:**
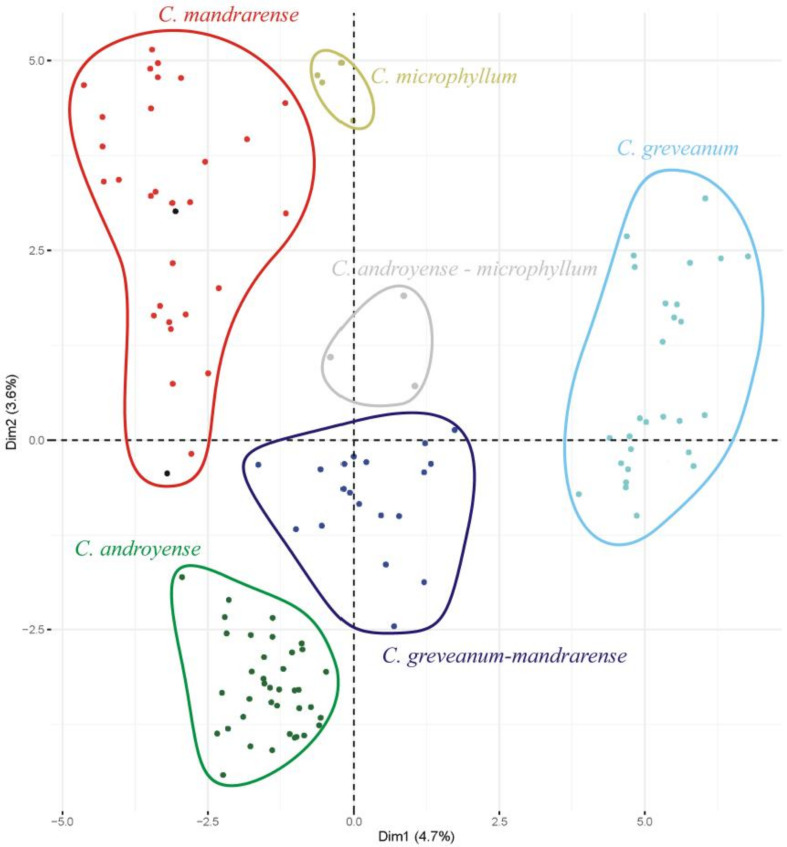
PCA scatter plot of the first two dimensions based on 22 morphological characters and 123 specimens. Black dots within the *Capurodendron mandrarense* cluster represent putative hybrids between that species and *C. androyense* (specimens 150 and 161 in Table 1).

Morphological ordination—The most important variables contributing to the axes were, in decreasing order of importance: petiole length (dimension 1: 8.8%, dimension 2: 11.1%), presence of hairs in the petiole (8.7%, 11.1%), leaf length (8.7%, 10.5%), presence of hairs in the current year’s shoots (8.1%, 9.5%), and leaf width (7.9%, 8.1%). Projections on axes 1 (4.7%) and 2 (3.6%) (Figure 1) show that *Capurodendron greveanum* is clearly different from the Arid Complex specimens. Within the complex, all morphospecies appear well delimited, but *Capurodendron microphyllum* can be divided into two groups, one containing the typical morphotype, the other with specimens displaying character states reminiscent of *C. androyense*. *Capurodendron androyense* and *C. mandrarense* are clearly separated on the plot, which contrasts with their genetic affinities (cf. below). Two specimens with intermediate morphologies between *Capurodendron androyense* and *C. mandrarense* appeared encompassed within the variability of *C. mandrarense* (black dots in Figure 1). The specimens corresponding to the *Capurodendron greveanum-mandrarense* morphotype are grouped together and are clearly separated from *C. greveanum.*

**Figure 2:**
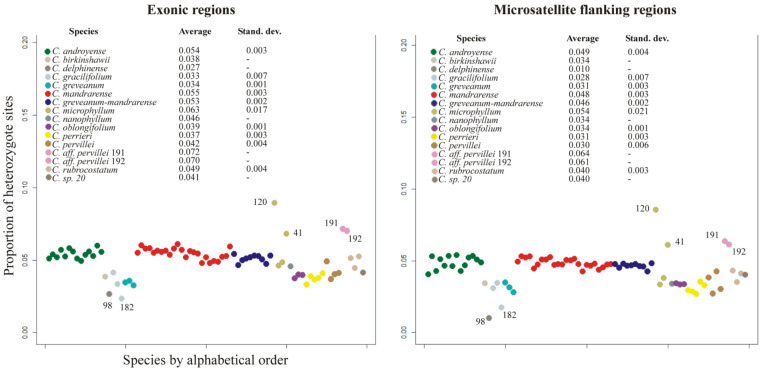
Proportion of heterozygous sites for exons (average 0.051, sd 0.013) and microsatellite flanking regions (0.045, sd 0.014), with average and standard deviation of each taxa indicated.

Heterozygosity—This value can theoretically range from 0 for complete homozygotes to 1 for complete heterozygotes. The average heterozygosity for the exonic dataset was 0.051 (standard deviation SD 0.013), while for microsatellite flanking regions it was 0.045 (SD 0.014). Both datasets provided the same pattern of heterozygosity (Figure 2), showing that heterozygous sites are not linked to coding or intergenic regions, but are evenly distributed throughout the whole genome. The lowest heterozygosity levels were found for *Capurodendron greveanum*, *C. gracilifolium*, *C. perrieri* and *C. oblongifolium* (≤0.033), while *C. microphyllum* was the species with the highest value, although with a high standard deviation (≥0.063) (Figure 2). Specimens 191 and 192, both belonging to *Capurodendron* aff. *pervillei*, showed the highest heterozygosity level after *C. microphyllum* specimen 120. The morphospecies *Capurodendron greveanum-mandrarense* did not show a higher heterozygosity than the other two species of the Arid Complex, *C. mandrarense* and *C. androyense*.

**Figure 3:**
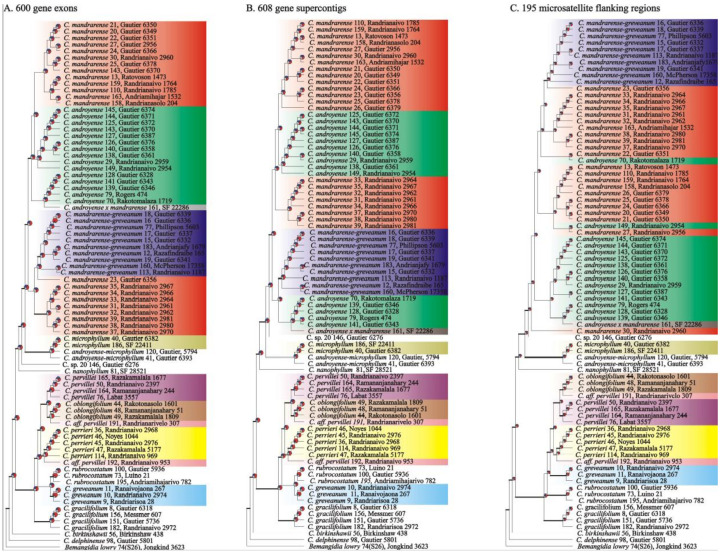
Pseudocoalescent ultrametric phylogenetic tree from Astral inferred from RAxML analyses of (**A**) 600 gene exonic regions, (**B**) 608 gene supercontigs including introns, exons and flanking regions and (**C**) 195 microsatellite flanking regions. All specimens contained less than 20% missing nucleotide positions. Colors refer to morphospecies. Pie charts represent the proportion of gene trees that support the clade of interest (red), support the main alternative bifurcation (blue), or support any other remaining alternative solution (gray). Astral posterior probabilities higher than 0.8 are depicted only for interspecific clades as bold lines. Species names are followed by the specimen number used in this study and the collector code.

Phylogenetic reconstructions—The three analyzed datasets (exonic regions, supercontigs, and microsatellite flanking regions) produced trees with a similar topology (Figure 3). The main difference between the three datasets were the positions of *Capurodendron* sp. 20 and *C. rubrocostatum*. *Capurodendron* sp. 20 is sister to the Arid Complex in the supercontig and microsatellite dataset, but sister to (Arid Complex + *C. microphyllum*) when using only exonic sequences. In the case of *Capurodendron rubrocostatum*, it is placed sister to (Western Complex + *C. greveanum*) in microsatellite flanking regions, but sister to *C. greveanum* in the remaining two datasets.

Species generally formed supported clades in at least one tree, except for the Arid Complex in which the three morphospecies *Capurodendron androyense*, *C. mandrarense* and *C. greveanum-mandrarense* appeared intermixed. Astral topologies without quartet scores, which are non-ultrametric (Figure S1), showed a radiation-like pattern in the Arid Complex, with all the main clades diverging from a single supported node. In the case of the Western Complex, the three described species appeared separated by long supported branches, but the two specimens of *Capurodendron* aff. *pervillei* are recovered polyphyletic and not sister to *C. pervillei*, but rather one to *C. perrieri* and the other to *C. oblongifolium*. Within *Capurodendron greveanum-mandrarense* morphospecies, specimens LG 6339, LG 6336 and Phillipson 5603, all collected from the same population, appeared tightly clustered at the tip of a long branch (Figure S1).

**Figure 4:**
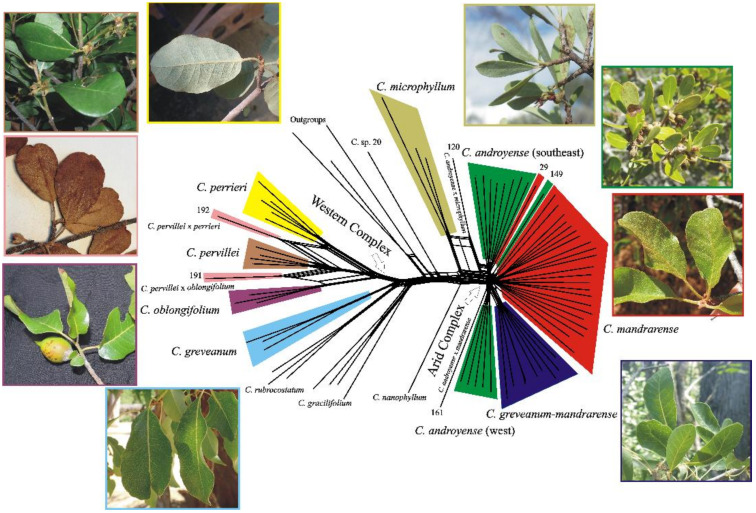
Split network computed from uncorrected P-distances and a concatenated supermatrix of exonic regions from 600 genes and 81 specimens. Splits and picture margin are color-coded according to morphospecies and specimens detected as hybrids are indicated. Numbers refer to specimens in Table 2. (Outgroups: *Bemangidia lowry*, *Capurodendron delphinense* and *C. birkinshawii*).

Split networks produced almost the same clusters for all three datasets (the exon dataset is presented in Figure 4), the only difference being found in the supercontig dataset, which produced the same topology but with longer branches for *Capurodendron pervillei*. *Capurodendron microphyllum* always appeared as a sister species to the Arid Complex, with specimen 120 quite isolated from all remaining ones. Samples from the Arid Complex produced a radiation-like pattern. *Capurodendron androyense* was split into three lineages, *C. mandrarense* into two, and *C. greveanum-mandrarense* was recovered as a single clade. The largest lineage of *Capurodendron androyense* was comprised only of southern specimens (S. Androy and SW Anosy), while the second largest group solely contained the southwestern specimens (surroundings of Toliara and Tsimanampetsotse NP). Within the Western Complex the three described species are monophyletic, but *Capurodendron* aff. *pervillei* appeared polyphyletic, with specimen 191 arising between *C. oblongifolium* and *C. pervillei* and specimen 192 between *C. perrieri* and *C. pervillei*.

Phasing—From the 638 gene-trees generated, only those based on alignments with more than 900 bp were taken into account (305 loci in total), as shorter alignments produced unsupported topologies. The trees were manually checked searching for genes displaying alleles clustered in different species, which can be considered a signal of hybridization. Specimens of *Capurodendron greveanum-mandrarense* always displayed alleles clustered within specimens of the Arid Complex, never with *C. greveanum*, and usually only with specimens of their own morphospecies.

In the *Capurodendron* Western Complex, each species displayed homologous alleles clustered together. However, the specimens *Capurodendron* aff. *pervillei* 191 and 192 contained 40% of the informative genes with homologous alleles nested into different species (Table 4). For the remaining 60% genes, both alleles were grouped together but sometimes in one species and sometimes in another: in *C. oblongifolium* and *C. pervillei* for specimen 191, and in *C. pervillei* and *C. perrieri* for 192.

**Microsatellites**—STRUCTURE output, using the ΔK method [49], suggested that our data best fit two gene pools for the Western Complex and three for the Arid Complex (Figure S2), however these numbers of clusters do not match well either with the phylogenetic species concept or with the morphological species concept.

In the Western Complex dataset, all specimens appeared completely admixed except the *Capurodendron pervillei* specimens 164 and 165, which are grouped together and without admixture. This pattern is stable from *k* = 2 to *k* = 10.

In the Arid Complex dataset, *Capurodendron greveanum* formed the most clearly isolated group at all *k* values. However, specimen 9 shared around 60% of its genetic component with the pool composed of *Capurodendron androyense* and *C. mandrarense* clusters, but not with *C. greveanum-mandrarense*. The second-best isolated pool, appearing from *k* = 3 and higher, was composed of the *Capurodendron greveanum-mandrarense* morphospecies, although specimens 19, 113, 160 and 183 displayed admixtures with *C. androyense* and *C. mandrarense*. *Capurodendron microphyllum* never appeared as a single gene pool, even at *k* = 10, nor did *C. androyense* and *C. mandrarense,* both of which displayed a highly intermixed pattern, except for specimens 31, 32, 33 and 34 of *C. mandrarense*, all collected from the same inland area of the Horombe plateau, at ca. 1000 m asl.

**Figure 5:**
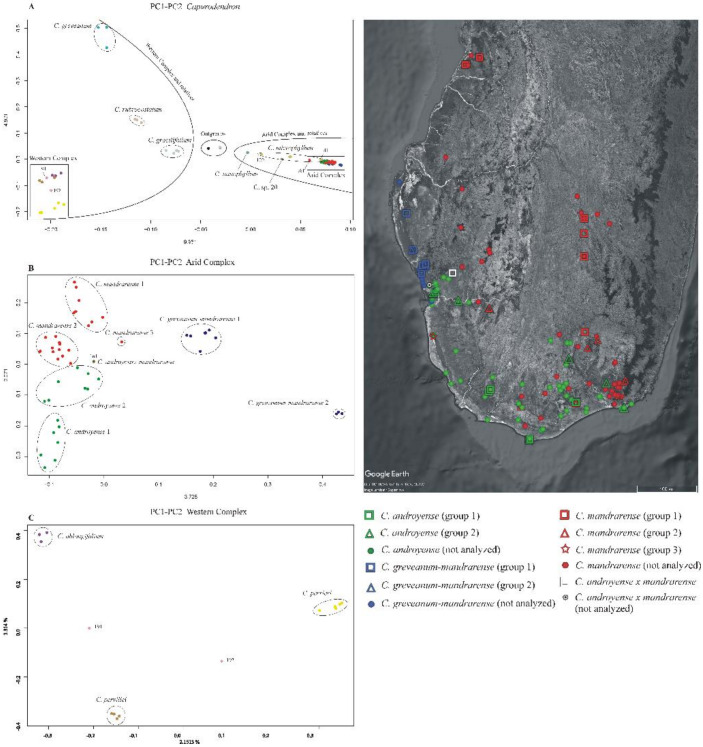
Principal Components Analysis (PCA) of 227 microsatellite flanking regions of (**A**) the complete dataset, (**B**) the Arid Species Complex, and (**C**) the Western Species Complex. The distribution map of the samples of the Arid Complex is shown on the righ side. Specimens 120 and 41 here included under *Capurodendron microphyllum*, share some morphological characters with *C. androyense*.

**Ordination of genetic data**—Analyses performed separately on exons, supercontigs and microsatellites flanking regions showed similar outputs and the same groups (Figure 5A). Axes information was always lower than 10%, which is expected when many markers and genetically closely related individuals are used. Three main clusters of dots were detected, one for the outgroup species, another for the Western Complex and related species (*Capurodendron gracilifolium*, *C. greveanum* and *C. rubrocostatum*), and one for the Arid Complex and related species (*C. microphyllum*, *C. nanophyllum* and *C.* sp. 20). Species outside the complexes were well delimited except for *Capurodendron microphyllum*, which showed a wide dot distribution (*C. nanophyllum* and *C.* sp. 20 are both known from a single specimen).

In the Arid Complex (Figure 5B), the PC1 axis clearly separated *Capurodendron greveanum-mandrarense* specimens from the rest, this morphospecies being further divided into two clusters, with group 2 containing only the three specimens that appeared on a long branch in the non-ultrametric Astral trees. The mutations that supported their differentiation from the other specimens were not caused by genome inversions nor by insertions/deletions, as the variable sites appeared dispersed throughout all loci. As mentioned above, all three specimens were collected from the same population, by two different collectors and in different years, and were processed and sequenced separately, which eliminates contamination as a possible explanation for the pattern.

All the remaining specimens share low values for PC1 and are scattered along the PC2 axis and could thus be considered as forming a large single group. However, the specimens identified as *Capurodendron androyense* were retrieved in the negative values whereas *C. mandrarense* were on the positive coordinates. *Capurodendron androyense* can be divided into two groups: group 1, with the more extreme PC2 values and composed of extreme Southern specimens (Androy and SW Atsimo-Andrefana regions); and group 2, including southwestern (Toliara surroundings and Tsimanampetsotse NP) and southeastern (S Anosy) specimens. *Capurodendron mandrarense* specimens can be split into three subgroups: group 1 containing the specimens from the northern half of the species distribution; group 2 from the southern half; and group 3, with a single specimen, from the extreme southwest in the Tsimanampetsotse NP. Specimen 161 retrieved between *Capurodendron androyense* and *C. mandrarense* on this projection; it was one of the two collected specimens that are morphologically intermediate between these two species, and was the only one that could be analyzed. 

In the Western Complex (Figure 5C) the three described species appeared completely isolated, forming three well-differentiated groups. Specimen *Capurodendron* aff. *pervillei* 191 was located halfway between *C. pervillei* and *C. oblongifolium*, while specimen *C.* aff. *pervillei* 192 was equidistant between *C. pervillei* and *C. perrieri*.

To calculate nucleotide diversity, pairwise FST and Patterson’s D statistics for the Arid Complex, 5 groups were used based on the results of the PCA, according to genetic affinities but also to geographical proximity of individuals, while keeping the number of specimens in each group as similar as possible (Figure 5). For *Capurodendron androyense*, groups 1 and 2 were analyzed separately, group 1 being the more variable, both genetically and geographically, although not all specimens can be considered as sympatric, and group 2 gathering sympatric specimens with either *C. mandrarense* or *C. greveanum-mandrarense*. For *Capurodendron mandrarense*, two groups were also used with individuals from group 2, from southern areas and growing in sympatry with either *C. androyense* or *C. greveanum-mandrarense*, and group 1 and 3 together, containing specimens from northern areas. For *Capurodendron greveanum-mandrarense* only group 1 was used for pairwise FST, as group 2 shows particularities that might introduce a bias into the analyses.

Nucleotide diversity showed slightly lower values for exons than for flanking SSR but with consistent results among them when the three morphospecies are taken into account. The highest values were those of *Capurodendron androyense* (0.484 ± 0.1110, 0.506 ± 0.1145) followed by *C. mandrarense* (0.477 ± 0.1086, 0.0505 ± 0.1106), and *C. greveanum-mandrarense* (0.0423 ± 0.1152, 0.0469 ± 0.1211). Within the different genetic groups from Figure 5, specimens in sympatry had higher values than specimens in allopatry or partial allopatry (*Capurodendron androyense* group 2, 0.492 ± 0.117, 0.514 ± 0.121 *vs* group 1, 0.456 ± 0.117, 0.481 ± 0.124 and *C. mandrarense* group 2, 0.473 ± 0.111, 0.503 ± 0.114 *vs* group (1+3), 0.455 ± 0.1180, 0.474 ± 0.119).

FST comparison (Table 5) showed similar values for exons and flanking STR. Overall the FST values were low, with the highest found between each group of morphospecies (between 0.139 and 0.094). Then the highest value was found between the two genetically most distant groups of *Capurodendron mandrarense* and *C. androyense* (*C. androyense* group 1 and *C. mandrarense* group 1; 0.090 for exons and 0.084 for flanking STR regions). The lowest FST were found between the two groups of *Capurodendron androyense* and *C. mandrarense* that are geographically in contact (*C. androyense* group 2 and *C. mandrarense* group 2; 0.024 and 0.025 for exons and flanking STR respectively).

D-statistics (Supplementary Materials Table S2) did not support any introgression between *Capurodendron greveanum* and *C. greveanum-mandrarense* or any of the other subgroups of the two other morphospecies of the Arid Complex. A low level of introgression (f4 ratio up to 0.08) between *Capurodendron microphyllum* and the two subgroups of *C. androyense* was detected, indicating that the introgression between the two species could predate the split of *C. androyense* in different groups. Introgression between the different subgroups of *Capurodendron androyense* and *C. mandrarense* was also detected, with the highest f4 ratio (0.48) found between the two sympatric populations, *C. androyense* group 2 and *C. mandrarense* group 2. However, Dmin score statistics were never significant for trios including these subgroups. Therefore, the sharing of derived alleles between trios is inconsistent with a single species-tree relating them, even in the presence of incomplete lineage sorting. Significant Dmin score only confirm introgression between *Capurodendron microphylum* and *C. androyense* group 1, group 2 and *C. mandrarense* group 2.

**Figure 6:**
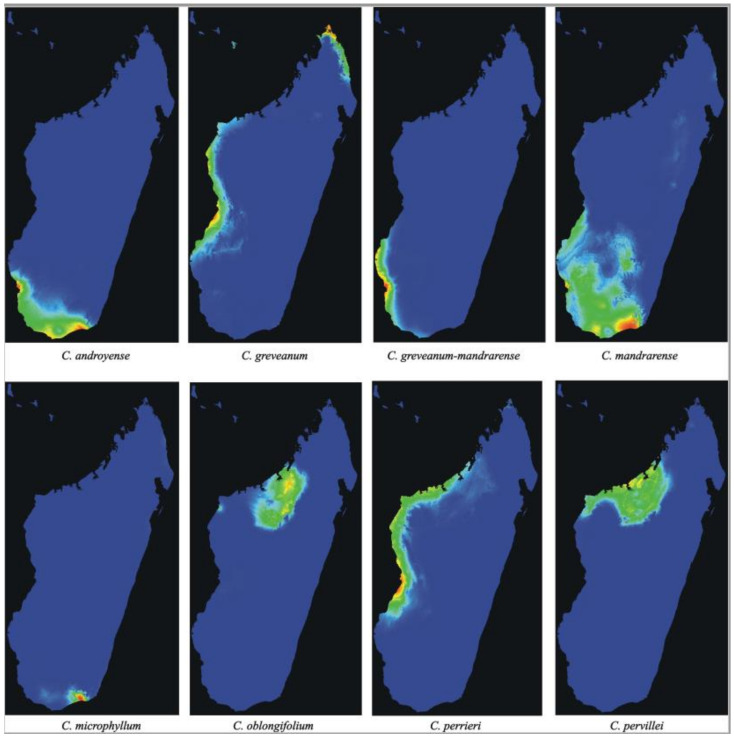
Potential distribution maps predicted by Maxent for *Capurodendron androyense*, *C. greveanum*, *C. greveanum-mandrarense*, *C. mandrarense*, *C. microphyllum*, *C. oblongifolium*, *C. perrieri* and *C. pervillei*.

**Analyses of potential distribution**—The potential distribution predicted by Maxent using 19 bioclimatic variables (Figure 6) showed AUC values of 0.99 for *Capurodendron androyense*, *C. greveanum-mandrarense*, *C. microphyllum*, *C. oblongifolium*, *C. perrieri* and *C. pervillei*, and 0.98 for *C. greveanum* and *C. mandrarense*, indicating a highly supported predicted distribution for all taxa. The three most significant bioclimatic variables contributing to the prediction of each species are shown in Table 6. The predicted distribution for *Capurodendron greveanum* shows two main areas with littoral or sublittoral conditions, one from Morombe (Menabe region) to Besalampy (Melaky region), and the other in the north-east, from Vohemar (SAVA region) to Antsiranana (DIANA region) separated by areas with unsuitable climatic conditions. These disjoint populations match with the species distribution according to specimen collections. *Capurodendron greveanum* cannot develop in the sub-arid regions of southern Madagascar where *C. androyense* grows, but its distribution meets the northwestern populations of *C. mandrarense*. It is only sympatric with *Capurodendron greveanum-mandrarense* in the Mangoky estuary, at the extreme south of its predicted distribution. Within the Arid Complex, each morphospecies shows different habitat preferences, with *Capurodendron androyense* tolerant of the driest habitats in the extreme southwest, and *C. mandrarense* preferring more humid places and extending to medium-altitudes, although overlapping with most of the distribution area of *C. androyense*. *Capurodendron greveanum-mandrarense* is apparently restricted to deciduous-forest habitats near the coast north of the Onilahy estuary, although it is also predicted further south down to Tsimanampetsotse, where it has never been collected. The regions closer to the sharp climatic gradient between dry spiny thicket and moist evergreen forests, just west of Taolagnaro (Fort-Dauphin), showed conditions suitable for *Capurodendron androyense*, *C. mandrarense* and *C. microphyllum*, where all these species have indeed been collected.

In the Western Complex, *Capurodendron perrieri* showed the most widespread distribution, occupying the coastal and near-coastal areas of western Madagascar. *Capurodendron oblongifolium* was restricted to inland north-western areas, while *C. pervillei* to inland and coastal north-western areas, partially in sympatry with *C. oblongifolium* and *C. perrieri*.

## 4. Discussion

**Ongoing speciation and the species concept**—The species concept is still a pending issue in biology, especially considering that species change across time. Among the 26 or so different species concepts proposed [50,51], those emphasizing species monophyly have been the most common in recent decades, due to the increasing use of genetic data. One of the most popular is that of de Queiroz [50], which defines a species as a lineage composed by a group of populations (a metapopulation) that evolves independently from others. This is also the definition that is most consistent with molecular phylogenies. However, this species concept sometimes fails at establishing the limits within taxonomically challenging groups, leading to mismatches between genetic groups and observed phenotypes [52,53]. This appears to be the case of the *Capurodendron* Arid Complex. 

Other proposals rely on a reference-based taxonomy [54], in which well-studied species limits of different living groups (e.g., humans/neanderthals/chimpanzees, in the case of primates) are used as references. Then, in a group under study, the decision that two lineages are different species would be taken if they show a similar or higher genetic differentiation than that of the two closest species in the reference. This could be done comparing, for example, FSTs between the reference and the candidate species under study. In the case of the Arid Complex, a comparison with deeply studied species-complexes such as sweet potato or *Citrus* [55,56] would perpetuate a mismatch between phenotypes and species obtained using such a concept, yielding a single species aggregating different morphologies without transitional forms.

Speciation can give rise to a new species from a parental one that persists unchanged, especially through founder effects, but also through hybridization, or when some subpopulations develop traits that increase their biological fitness and allow a fast adaptation to a different niche [57,58,59]. In such cases, the new species is monophyletic, but still nested within the parental one, resulting in a paraphyletic taxon. Although ecologically and phenotypically well-defined species can originate from a reduced number of key mutations in regulatory genes, the monophyletic species concepts will never recognize a new species as long as it is nested within the parental taxa [60,61]. Strong or even weak reproductive barriers between the two recently diverging lineages will produce differential accumulation of mutations across time, allowing a monophyletic species concept to eventually recognize two distinct species. However, if the isolation is not strong enough, the diverging lineages will hybridize, resulting in the introgression of genetic material of one species into the other and then leading to the termination of the speciation event.

Rather than the two species concepts mentioned above, each unsatisfactory for lineages in a speciation/introgression process, a population genetics approach in which species can be considered as historically connected populations sharing similar phenotypes and roles in the ecosystem brings together the temporal and phenotypic dimensions of species. This concept is developed by Freudenstein et al. [62] and is compatible with paraphyletic lineages sharing the same phenotype, and accounts for one of the most common problems in species delimitation during speciation processes. This is the concept we will use hereafter for well-characterized morphospecies lacking genetic isolation.

Species delimitation in the *Capurodendron* Western Complex—This species complex forms a well-defined group, sister to *Capurodendron rubrocostatum* and *C. greveanum*. It contains three morphologically and genetically delimited species (*Capurodendron oblongifolium*, *C. perrieri* and *C. pervillei*), and the unique taxonomic problem that we faced was to determine whether specimens 191 (Randrianarivelo 307) and 192 (Randrianaivo 953), both displaying morphologies similar to *C. pervillei*, should be considered a new species. 

Observed heterozygosities in both specimens are much higher than statistically expected (Figure 2); they contain the highest proportion of heterozygous sites across all studied *Capurodendron* specimens with the exception of *C. microphyllum* 120 (Gautier 5794). It is well known that hybridization increases the number of heterozygous sites, as each allele comes from a different taxon with a different history of mutations [63]. Phylogenetic networks (Figure 4) show how these specimens arise from lineages belonging to two different species, indicating that a great proportion of the variable sites are not specific to them, but are shared more or less in the same proportion with the parental taxa. The same conclusion holds for PCA analysis (Figure 6), with both specimens located halfway between their putative parental species. It can thus be assumed with confidence that they are of hybrid origin. However, contrary to what could have been expected, STRUCTURE results on microsatellites were unable to show either species isolation or introgression signals on both hybrid specimens. This may be the result of loci selection for gene capture, as phylogenetically distant species were used to design the probes and the resulting microsatellites might not be sufficiently informative. This limitation has also been observed in other groups of *Capurodendron* [14].

The phased phylogenetic gene trees (Table 4) are also key results supporting a hybrid origin: in many genes trees, the two alleles of specimens 191 and 192 are each nested in one of the parental species, a feature never observed with the alleles of the three recognized species of the complex. In a first-generation hybrid, it is expected that half the alleles will appear nested in one species, and the other half nested in the other. However, this proportion can be skewed by events such as shared or uninformative mutations, and can also be biased by phylogenetic reconstruction methods. This balanced pattern furthermore quickly disappears when the hybrid backcrosses with the parental species or reproduces with other hybrids, since introgression from one of the taxa will increase and recombination may recover parental chromosome blocks [64]. Phylogenetic reconstructions might be biased, at least for some genes, especially if parental species are close and if sequences code for proteins. In our case, specimens 191 and 192 may correspond to first generation (F1) hybrids, as the introgression of both parental species is quite balanced. The slightly skewed pattern observed for both samples might be due to the fact that many alleles could not be attributed to a given parental species because they were not informative enough. However, the striking coincidence of the skewness in the two hybrids (42.6% toward *C. pervillei*) is noteworthy. We could not find any reason for this other than the influence of incomplete lineage sorting (ILS) among the parental species.

Although flowers were observed on these hybrid specimens, it is not clear whether they are fertile or sterile, as fruits, even in the first developmental stages, were absent. The parental species are phylogenetically close, which would suggest that hybrids could be fertile. However, if hybridization yielding fertile offspring were a recurrent process in the group, the species of the Western Complex would be expected to be more intermixed genetically, a pattern that was not detected in our analyses (Figure 2, Figure 3, Figure 4 and Figure 5). From the 91 specimens studied morphologically, just two contained a phenotype different from any described species of the complex, indicating that morphological intermediates are rare and hence probably sterile. However, it is also possible that hybrids are indeed fertile, but that their offspring are not fit in this environment and thus removed by natural selection.

Our data clearly indicate that both specimens correspond to hybrids and should therefore receive the name *Capurodendron pervillei* x *oblongifolium* for 191 (Randrianarivelo 307), and *C. pervillei* x *perrieri* for 192 (Randrianaivo 953). Consistent with this, both specimens were collected in areas where the parental species coexist. The *Capurodendron* Western Complex is therefore composed of three well-differentiated species that can hybridize. Each one needs to be assessed for conservation separately, without inclusion of the hybrids. Hybrids do not require descriptions nor conservation assessment.

Species delimitation in the *Capurodendron* Arid Complex—This species complex forms a well-supported lineage closely related to *Capurodendron microphyllum*. Three morphospecies can be easily distinguished with the naked eye, and they are supported by morphological analysis (Figure 1, red, green, and dark blue). Only two of the 123 specimens studied (1.6%) showed intermediate phenotypes: specimens 150 (SF 22230) and 161 (SF 22286). Genetic analyses, however, show that the three morphospecies are entangled, despite clear morphological discontinuity and different environmental preferences (although with partly sympatric distributions; Figure 6). Since we used loci ranging from low to high substitution rates, even reaching geographical resolution, (Figure 5B) we can conclude that the absence of monophyly is not an artefact produced by non-appropriate loci.

The morphospecies *Capurodendron greveanum-mandrarense* was initially hypothesized as a hybrid between the species referred to in its provisional name [1,14], but no signal supporting this hypothesis has been found so far. High heterozygosity levels (such as the ones observed in the Western Complex) are linked to recent hybridizations, yet in this morphospecies the heterozygosity level is even lower than that found in the other morphospecies of the complex (Figure 2). Phylogenetic networks are suitable diagrams for representing evolutionary relationships in groups that have experienced reticulation [65] and are useful in identifying ambiguous relationships [66]. Applied to our data, hybridization in the Western Complex as well as between *Capurodendron androyense* and *C. microphyllum* was clearly visible (Figure 4). However, no such signal could be observed for *Capurodendron greveanum-mandrarense*, which always constitutes a well-defined clade nested within the Arid Complex (Figure 3 and Figure 4). STRUCTURE on STR separates this morphospecies from *Capurodendron greveanum* even at *k* 2 and shows no admixture within both taxa (Figure S1). PCA (Figure 5B) clearly separates *C. greveanum-mandrarense* from the remaining specimens of the complex and confirms its location far from *C. greveanum*. Finally, phased phylogenetic reconstructions showed no alleles coming from *Capurodendron greveanum*. Thus, *Capurodendron greveanum-mandrarense* is not a hybrid between *C. greveanum* and *C. mandrarense*, but rather an undescribed species displaying morphological convergence with both, and more related to the latter. The differentiation of this species might relate to a local adaptation to the less arid, sandy and coastal conditions found just North of Toliara, limiting its further expansion. It requires formal description and a conservation assessment of its own, and will be referred to hereafter as *Capurodendron mikeorum* nom. prov., as it grows in the forest harboring the Mikea ethnical group.

Conversely, the two morphospecies *Capurodendron androyense* and *C. mandrarense* are not only genetically unresolved, they also display a higher genetic diversity in areas where they grow sympatrically (Figure 5). Two non-exclusive hypotheses can explain this pattern:

i. An ongoing sympatric speciation: Although the morphospecies have a widely overlapping distribution, they show different environmental preferences (Figure 6). *Capurodendron androyense* is the more drought-resistant taxon, extending to the areas with 12 ecologically dry months found along the coast in the extreme south, while *C. mandrarense* prefers relatively more humid habitats, is more cold-tolerant, and has a distribution extending to south-central regions up to 1000 m elevation. Hence, a partially ongoing sympatric speciation mediated by environmental selection might be at work. In such a case, the area with the highest nucleotide diversity, which coincides with the genetic clusters *Capurodendron androyense* 2 and *C. mandrarense* 2 (Table 5; Figure 5) representing the majority of the complex distribution area, could correspond to the diversification center. This is a climatically intermediate area with two less months of dry season than the coastal region, while also escaping the colder night temperatures of the central highlands. From this region, the ancestral species could have undergone a selection pressure towards aridity (enforcing the *Capurodendron androyense* gene pool), and towards more humid and colder habitats (enforcing the *C. mandrarense* gene pool). Then, *Capurodendron androyense* 1 and *C. mandrarense* 1 of Figure 5 would have appeared later, having many fewer introgression signals between them and being therefore genetically ‘purer’. This pattern would correspond to a parapatric speciation process driven by ecological adaptation. In such a scenario of recent speciation, it is not surprising to observe such levels of incomplete lineage sorting (ILS), as the two species are additionally found in large areas and are expected to have high population effective sizes [67].
ii. A past allopatric speciation followed by secondary contact: Under this hypothesis the species would have originated in allopatry from a recent common ancestor, adapting to different environments. Posteriorly, *Capurodendron androyense* and *C. mandrarense* distributions would have expanded and come into contact, producing the more admixed *C. androyense* group 2 and *C. mandrarense* group 2. In this scenario, the higher nucleotide diversity of these groups would point to a secondary contact with introgression rather than to an ancient center of diversification. This would parallel a similar situation to the east, where hybrids between *Capurodendron androyense* and *C. microphyllum* (a genetically well differentiated species, sister to the Arid Complex) appear in areas where both taxa coexist. In this case, ILS signal would come from admixture.

Independently of which hypothesis corresponds to what actually occurred (the situation could be more complex still, involving a combination of both), the three morphospecies can coexist in the same forest without forming a population of phenotypically intermediate hybrid specimens. This indicates that selection pressure is keeping each morphospecies separate, and as such, they merit a taxonomical rank. Genetic similarity would tend to support an infraspecific level, such as subspecies. However, a species rank seems more appropriate phenotypically, as more differences are found between these entities than among many other clearly separated *Capurodendron* species. Accordingly, we prefer to use the species concept of Freudenstein et al. [62] and consider each of the three morphospecies of the Arid Complex as valid species: *Capurodendron androyense*, *C. mandrarense* and *C. mikeorum* nom. prov., each deserving a conservation assessment.

Potentials and limitations of genetic data for species delimitation and conservation: lessons from our case study—When implementing the Freudenstein et al. [62] species concept, characterization basically relies on morphology, giving a minor contribution to the genetic analyses we performed. In fact, species identification is simpler by visualization with the naked eye than relying on genetic data. Nonetheless, morphological species delimitation has to be validated by molecular analyses such as the ones we implemented here in order to discard the hypothesis that the morphospecies is the result of recent hybridization.

If a strict morphological species concept had been applied in the Western Complex, a new species (corresponding to *Capurodendron* aff. *pervillei* samples) should have been described. However, genetic data clearly demonstrated that these samples corresponded to sporadic hybrids that do not deserve species recognition. Additionally, since we found a clear parallel between morphology and genetics across the three species of this complex, it seems that the observed hybridization represents sporadic events with little consequence for parental species integrity. Similarly, in the Arid Complex, the genetic analyses conducted on a large number of highly variable loci allowed us to separate the three morphospecies that represent true species (including an undescribed one), from those that should be considered hybrids (*Capurodendron androyense x microphyllum* and *C. androyense x mandrarense*). In the absence of such genetic data, *Capurodendron androyense x microphyllum* specimens indeed could have been erroneously interpreted as representing a plain species. Furthermore, *Capurodendron androyense x mandrarense* specimens might have been considered evolutionary intermediates between *C. androyense* and *C. mandrarense*, an interpretation that would put the latter two species into question. The former hybrids do not merit a species rank even when using the Freudenstein concept, as it appears they need their parental species in order to persist. Conversely, without our molecular analyses excluding a hybrid origin, *Capurodendron mikeorum,* nom. prov. (referred to initially as *C. greveanum-mandrarense*) would have been wrongly interpreted as a nothospecies, instead of a plain species. 

Here, four analyses were critical in detecting hybridization: PCA, calculation of heterozygosity levels, reconstruction of phased phylogenies, and reconstruction of phylogenetic networks. From these, the last one provided the best ratio working time/output fidelity, whereas PCA is only conclusive if combined with others. Phasing was the most powerful tool to reveal recent hybrids and their parental species. 

The IUCN Red List of Threatened Species ([68,69] IUCN, 2004) is a practical tool allowing the rapid accumulation of results for a broad panel of organisms while providing incentives for additional conservation measures, for example at regional and national levels [70]. However, the underlying assumption is that the organisms assessed are well delimited species with little or no genetic exchange between them. Although this may be the case with the majority of taxa, there nevertheless is a portion for which this is not the case, including the complexes studied here. To accommodate these situations, the IUCN allows conservation assessments on subspecific ranks and even geographically remote subpopulations (https://www.iucnredlist.org/resources/tax-sources, accessed on 12 August 2021). As a consequence, considering each of the morphospecies of the Arid Complex either as species or alternatively as subspecific taxa should have no impact on their conservation. 

If we aim at preserving the genetic diversity within the species as accepted here, we should address the subpopulations separately. The Arid Complex contains lineages genetically ‘purer’ than others in both *Capurodendron androyense* and *C. mandrarense*. Genetically pure populations may deserve more attention compared to admixed ones. However, the impact of admixture on species fitness and genetic diversity is globally not well understood, and certainly not for *Capurodendron*. As long as we are unsure whether the observed admixture is ancestral or caused by secondary contact (resolving this would imply other methodologies involving population genetics modeling [71], we cannot decide if this phenomenon is increasing or curtailing genetic diversity. Furthermore, these populations, despite being partially delimited geographically, can only be circumscribed using genomic tools. As a consequence, genetic assessment of subpopulations is impracticable and their management almost impossible in the field.

As assessments of hybrids are not recommended for the IUCN Red list (except plant hybrids treated as species), the morphospecies *Capurodendron androyense* x *mandrarense*, *C. androyense* x *microphyllum*, *C. pervillei* x *oblongifolium*, and *C. pervillei* x *perrieri* do not need a conservation status. From a conservation point of view, the role of hybrids in population dynamics is unclear [72,73,74]. On the one hand, they could favor species extinction due to the introgression of one taxon in another, leading, for example, to a reduction of the populations of *Capurodendron microphyllum*, which would be partially replaced by *C. androyense x microphyllum*. On the other hand, hybridization may produce recurrent introgression of genes able to increase and diversify population richness, allowing greater resilience of a species to climatic change or other biological factors.

Conservation assessments—*Western complex—*species conservation assessments for *Capurodendron perrieri* (Near Threatened, NT; [75]) and *C. pervillei* (NT; [76]) have been already published and are not affected by the results obtained here. *Capurodendron oblongifolium* is a recently described species [1] for which we propose the following provisional conservation assessment:

*Capurodendron oblongifolium*: The Extent of Occurrence (EOO) is estimated to be 2024 km^2^ and the Area of Occupancy (AOO) 24 km^2^; the species is documented from five locations with respect to the most plausible threat which is habitat destruction due to uncontrolled forest fires, one location being outside the protected area network. With low values in AOO, EOO and one location outside the protected area network in a region regularly impacted by forest fires, continuing decline is projected and the species is preliminarily assessed as Endangered (EN B1ab(i,ii,iii,iv)+2ab(i,ii,iii,iv)).

*Arid Complex—*The conservation assessment of *Capurodendron androyense* was previously assessed as Least Concern (LC; [77]), however its evaluation included a subpopulation recognized here as *C. mandrarense* as well as the specimens identified here as *C. androyense x microphyllum*, which should be excluded. Their exclusion does not alter the Least Concern status of this species, but EOO and AOO values have been recalculated and are now 44,052 and 244 km^2^, respectively. In the case of *Capurodendron mandrarense*, the difficulty differentiating it from the *C. greveanum-mandrarense* specimens (= *C. mikeorum* nom. prov.) complicated its evaluation, leaving this taxon as data deficient. Finally, *Capurodendron mikeorum* nom. prov. should be considered a valid species. Preliminary conservation assessments for these two taxa are proposed below.

*Capurodendron mandrarense*: This species occurs in southern and southwestern Madagascar. The estimated extent of occurrence (EOO) calculated with all available collections is 120,210 km^2^, and the minimum area of occupancy (AOO) is 244 km^2^ (qualifying for Endangered under criterion B2). It is known from 78 collections, from 22 locations, 16 outside the protected areas network. Threats include agriculture expansion, selective logging, charcoal production and uncontrolled forest fires. Despite a projected continuing decline, at least outside protected areas, this species cannot be considered severely fragmented and is here assessed as Least Concern.

*Capurodendron mikeorum nom. prov*: This species is restricted to sandy soils from the south-west of Madagascar. The estimated extent of occurrence (EOO) calculated with all available herbarium specimen data is 1676 km^2^, and the minimum area of occupancy (AOO) is 72 km^2^ (both qualifying for EN under criterion B). The species is known from 24 herbarium collections from merely five locations, and faces threats from large-scale agriculture, uncontrolled forest fires, and selective logging. Despite the five locations being in or near protected areas, the pressures facing dry forests in the southwestern part of Madagascar, even in protected areas, point toward a continued decline, justifying assigning this species to the category Endangered (B1ab(i,ii,iii,iv,v)+2ab(i,ii,iii,iv,v)).

## Figures and Tables

**Table 1:** Morphospecies included in each species complex with information related to their delimitation, distribution and phylogenetic status.

	Morphospecies	Details
Western Complex	*C. oblongifolium*	Well delimited, >50 km inland, sympatric with *C. pervillei.*
	*C. perrieri*	Well delimited, <50 km from the coast, sympatric with *C. pervillei.*
	*C. pervillei*	Well delimited, widespread and sympatric with both *C. oblongifolium* and *C. perrieri.*
	*C. aff. pervillei*	Similar to *C. pervillei*, not monophyletic, rare occurrences scattered in the global area of the complex.
Arid Complex	*C. androyense*	Widespread, well delimited morphologically, except three specimens intermediate with *C. microphyllum* and two with *C. mandrarense.*
	*C. greveanum-mandrarense*	Restricted range N of Toliara, weakly delimited morphologically, characters shared with *C. greveanum* and *C. mandrarense*.
	*C. mandrarense*	Widespread, variable, weakly differentiated from *C. greveanum-mandrarense* but more hairy and with prominent nerves. Two specimens intermediate with *C. androyense*.
Similar species	*C. greveanum*	Widespread in two disjunct coastal populations. Weakly differentiated from *C. greveanum-mandrarense*, but completely glabrous vegetatively. Phylogenetically far from the Arid Complex.
	*C. microphyllum*	Restricted range W of Fort-Dauphin, well delimited except three specimens intermediate with *C. androyense*. Sister species to the Arid Complex.

**Table 2:** Information on the specimens used. Original identification, followed by collector’s name and number, collection year and sample kind.

Lab. Code	Morphospecies	Region	Collector Code	Year	Origin ^1^
128	*Capurodendron androyense*	Atsimo-Andrefana	Gautier 6328	2017	Silica gel (G)
141	*C. androyense*	Atsimo-Andrefana	Gautier 6343	2017	Silica gel (G)
139	*C. androyense*	Atsimo-Andrefana	Gautier 6346	2017	Silica gel (G)
140	*C. androyense*	Atsimo-Andrefana	Gautier 6358	2017	Silica gel (G)
138	*C. androyense*	Atsimo-Andrefana	Gautier 6361	2017	Silica gel (G)
143	*C. androyense*	Androy	Gautier 6370	2017	Silica gel (G)
144	*C. androyense*	Androy	Gautier 6371	2017	Silica gel (G)
125	*C. androyense*	Androy	Gautier 6372	2017	Silica gel (G)
145	*C. androyense*	Androy	Gautier 6374	2017	Silica gel (G)
126	*C. androyense*	Androy	Gautier 6376	2017	Silica gel (G)
127	*C. androyense*	Anosy	Gautier 6387	2017	Silica gel (G)
149	*C. androyense*	Anosy	Randrianaivo 2954	2017	Silica gel (G)
29	*C. androyense*	Anosy	Randrianaivo 2959	2017	Silica gel (G)
79	*C. androyense*	Atsimo-Andrefana	Rogers 474	2004	G
70	*C. androyense*	Atsimo-Andrefana	Rakotomalaza 1719	1998	G
150	*C. androyense-mandrarense*	Atsimo-Andrefana	SF 22230	1962	G
161	*C. androyense-mandrarense*	Atsimo-Andrefana	SF 22286	1962	G
56	*C. birkinshawii*	Anosy	Birkinshaw 438	1997	G
98	*C. delphinense*	Anosy	Gautier 5801	2011	Silica gel (G)
151	*C. gracilifolium*	Melaky	Gautier 5736	2011	Silica gel (G)
8	*C. gracilifolium*	Atsimo-Andrefana	Gautier 6318	2017	Silica gel (G)
182	*C. gracilifolium*	Menabe	Randrianaivo 2972	2017	Silica gel (G)
156	*C. gracilifolium*	Atsimo-Andrefana	Messmer 607	1998	G
9	*C. greveanum*	DIANA	Randriarisoa 28	2017	Silica gel (G)
11	*C. greveanum*	Atsimo-Andrefana	Ranaivojaona 267	2000	G
10	*C. greveanum*	Menabe	Randrianaivo 2974	2017	Silica gel (G)
163	*C. mandrarense*	Anosy	Andriamihajarivo 1532	2004	G
20	*C. mandrarense*	Atsimo-Andrefana	Gautier 6349	2017	Silica gel (G)
21	*C. mandrarense*	Atsimo-Andrefana	Gautier 6350	2017	Silica gel (G)
22	*C. mandrarense*	Atsimo-Andrefana	Gautier 6351	2017	Silica gel (G)
23	*C. mandrarense*	Atsimo-Andrefana	Gautier 6356	2017	Silica gel (G)
24	*C. mandrarense*	Androy	Gautier 6366	2017	Silica gel (G)
25	*C. mandrarense*	Androy	Gautier 6378	2017	Silica gel (G)
26	*C. mandrarense*	Androy	Gautier 6379	2017	Silica gel (G)
158	*C. mandrarense*	Anosy	Randrianasolo 204	1991	G
13	*C. mandrarense*	Anosy	Ratovoson 1473	2008	P
159	*C. mandrarense*	Anosy	Randrianaivo 1764	2009	G
110	*C. mandrarense*	Anosy	Randrianaivo 1785	2011	G
27	*C. mandrarense*	Anosy	Randrianaivo 2956	2017	Silica gel (G)
30	*C. mandrarense*	Anosy	Randrianaivo 2960	2017	Silica gel (G)
31	*C. mandrarense*	Anosy	Randrianaivo 2961	2017	Silica gel (G)
32	*C. mandrarense*	Anosy	Randrianaivo 2962	2017	Silica gel (G)
33	*C. mandrarense*	Anosy	Randrianaivo 2964	2017	Silica gel (G)
34	*C. mandrarense*	Ihorombe	Randrianaivo 2966	2017	Silica gel (G)
35	*C. mandrarense*	Ihorombe	Randrianaivo 2967	2017	Silica gel (G)
37	*C. mandrarense*	Menabe	Randrianaivo 2970	2017	Silica gel (G)
38	*C. mandrarense*	Menabe	Randrianaivo 2980	2017	Silica gel (G)
39	*C. mandrarense*	Menabe	Randrianaivo 2981	2017	Silica gel (G)
162	*C. mandrarense*	Ihorombe	SF 6692	1952	G
183	*C. mandrarense-greveanum*	Atsimo-Andrefana	Andrianjafy 1679	2006	P
15	*C. mandrarense-greveanum*	Atsimo-Andrefana	Gautier 6332	2017	G
16	*C. mandrarense-greveanum*	Atsimo-Andrefana	Gautier 6336	2017	G
17	*C. mandrarense-greveanum*	Atsimo-Andrefana	Gautier 6337	2017	G
18	*C. mandrarense-greveanum*	Atsimo-Andrefana	Gautier 6339	2017	G
19	*C. mandrarense-greveanum*	Atsimo-Andrefana	Gautier 6341	2017	G
160	*C. mandrarense-greveanum*	Atsimo-Andrefana	McPherson 17358	1998	G
77	*C. mandrarense-greveanum*	Atsimo-Andrefana	Phillipson 5603	2002	G
12	*C. mandrarense-greveanum*	Atsimo-Andrefana	Razafindraibe 165	2006	G
113	*C. mandrarense-greveanum*	Atsimo-Andrefana	Randrianaivo 1187	2005	G
40	*C. microphyllum*	Anosy	Gautier 6382	2017	Silica gel (G)
186	*C. microphyllum*	Anosy	SF 22411	1963	G
120	*C. microphyllum-androyense*	Anosy	Gautier 5794	2011	Silica gel (G)
41	*C. microphyllum-androyense*	Anosy	Gautier 6393	2017	Silica gel (G)
81	*C. nanophyllum* (Type)	Androy	SF 28521	1968	G
46	*C. perrieri*	Menabe	Noyes 1044	1992	G
47	*C. perrieri*	Atsimo-Andrefana	Razakamalala 5177	2010	G
114	*C. perrieri*	Boeny	Randrianaivo 969	2003	G
36	*C. perrieri*	Menabe	Randrianaivo 2968	2017	Silica gel (G)
45	*C. perrieri*	Menabe	Randrianaivo 2976	2017	Silica gel (G)
190	*C. oblongifolium*	Boeny	PerrierBâthie 1105	1974	P
44	*C. oblongifolium*	Sofia	Rakotonasolo 1601	2015	G
48	*C. oblongifolium*	Sofia	Ramananjanahary 51	2004	G
49	*C. oblongifolium*	Sofia	Razakamalala 1809	2004	G
76	*C. pervillei*	Boeny	Labat 3557	2005	G
164	*C. pervillei*	Sofia	Ramananjanahary 244	2004	G
165	*C. pervillei*	Sofia	Razakamalala 1677	2004	G
50	*C. pervillei*	Sofia	Randrianaivo 2397	2013	G
191	*C. aff. pervillei*	Boeny	Randrianarivelo 307	2005	G
192	*C. aff. pervillei*	Boeny	Randrianaivo 953	2003	G
195	*C. rubrocostatum*	Boeny	Andriamihajarivo 782	2005	G
194	*C. rubrocostatum*	Atsimo-Andrefana	Chauvet 187	1961	G
100	*C. rubrocostatum*	Melaky	Gautier 5936	2012	Silica gel (G)
73	*C. rubrocostatum*	Melaky	Luino 21	2012	G
146	*C. sp.* 20	Boeny	Gautier 6276	2016	Silica gel (G)
74(S26)	*Bemangidia lowry*	Anosy	Gautier 5789	2011	Silica gel (G)

^1^ If sampled from a herbarium specimen, then the herbarium code; if sampled in the field, then “Silica gel”.

**Table 3:** Characters used for the principal coordinate analysis with 123 specimens of the *Capurodendron* Arid Complex.

Character Number	Character	Type	Coding	State
1	Plant height	Continuous	meters	Number of meters
2	Brachyblast	Discrete	0	Absent
			1	Present
3	Prior year’s elongating shoots	Discrete	0	Green and glabrous
			1	Brown and hairy
4	Petiole length	Continuous	mm	Number of mm
5	Petiole hairs	Discrete	0	Glabrous
			1	With hairs
			2	Tomentose
6	Leaf base symmetry	Discrete	0	Symmetric
			1	Asymmetric
7	Leaf base	Discrete	0	Decurrent
			1	Cuneate
			2	Obtuse
			3	Subcordate
8	Leaf length	Continuous	mm	Number of mm
9	Leaf width	Continuous	mm	Number of mm
10	Broadest leaf region	Discrete	0	1st third
			1	2nd third
			2	3rd third
11	Leaf apex	Discrete	0	Acute
			1	Obtuse
			2	Rounded
			3	Emarginated
12	Leaf upper side hairs	Discrete	0	Glabrous
			1	With hairs
			2	Tomentose
13	Leaf lower side hairs	Discrete	0	Glabrous
			1	With hairs
			2	Tomentose
14	Midrib on the lower side	Discrete	0	Not prominent
			1	Prominent
15	Secondary nerves on the lower side	Discrete	0	Not prominent
			1	Prominent
16	Midrib hairs on the upper side	Discrete	0	Glabrous
			1	With hairs
			2	Tomentose
17	Midrib hairs on the lower side	Discrete	0	Glabrous
			1	With hairs
			2	Tomentose
18	Pairs of secondary nerves	Continuous	Number	Number of pairs
19	Pedicel length of flower	Continuous	mm	Number of mm
20	Calyx hairs	Discrete	0	Adpressed
			1	Hirsute
21	External sepal length	Continuous	mm	Number of mm
22	Calyx diameter	Continuous	mm	Number of mm

**Table 4:** Topological positions of the alleles of specimens 191 (Randrianarivelo 307) and 192 (Randrianaivo 953) based on 305 maximum likelihood trees from protein coding genes. Monospecific loci refer to loci in which both alleles appeared nested in a single species, while heterospecific loci point to exons with each allele nesting within distinct species. Percentages are calculated after having discarded any missing or unsupported allele.

	Specimen 191	Specimen 192
Alleles in *C. oblongifolium*	192 (56.8%)	2 (0.7%)
Alleles in *C. perrieri*	2 (0.6%)	160 (56.7%)
Alleles in *C. pervillei*	144 (42.6%)	120 (42.6%)
Missing/unsupported alleles	137	164
Monospecific loci	101 (59.7%)	85 (60.3%)
Heterospecific loci	68 (40.3%)	56 (39.7%)

**Table 5:** FST values (weighted mean and standard deviation) within the Arid complex for the *Capurodendron* groups shown in Figure 5. The groups that were not compared are indicated with the symbol ‒.

Exons Flanking Regions FST						
	*C. androyense*	*C. androyense* 1	*C. androyense* 2	*C. mandrarense*	*C. mandrarense* 1	*C. mandrarense 2*
*C. androyense* 1	‒					
*C. androyense* 2	‒	0.037 ± 0.073				
*C. mandrarense*	0.033 +− 0.052	‒	‒			
*C. mandrarense* 1	‒	0.090 +− 0.117	‒	‒		
*C. mandrarense* 2	‒	‒	0.024 ± 0.058	‒	0.046 ± 0.074	
*C. greveanum-mandrarense*	0.118 ± 0.114	0.139 ± 0.142	0.095 ± 0.110	0.104 ± 0.111	0.111 ± 0.122	0.111 ± 0.122
**STR Flanking Regions FST**						
	*C. androyense*	*C. androyense* 1	*C. androyense* 2	*C. mandrarense*	*C. mandrarense* 1	*C. mandrarense* 2
*C. androyense* 1	‒					
*C. androyense* 2	‒	0.037 ± 0.079				
*C. mandrarense*	0.033 ± 0.052	‒	‒			
*C. mandrarense* 1	‒	0.084 ± 0.117	‒	‒		
*C. mandrarense* 2	‒	‒	0.025 ± 0.063	‒	0.044 ± 0.081	
*C. greveanum-mandrarense*	0.112 ± 0.112	0.122 ± 0.140	0.094 ± 0.120	0.108 ± 0.113	0.111 ± 0.139	0.097 ± 0.122

**Table 6:** The most important variables contributing to the potential distribution of each species. AUC = Area below the curve.

Species	Number of Points	Most Important Variables	Jackknife of AUC
*Capurodendron androyense*	90	Precipitation of wettest month	0.94
		Precipitation of wettest quarter	0.93
		Annual precipitation	0.92
*Capurodendron greveanum*	79	Annual precipitation	0.94
		Annual mean temperature	0.92
		Mean temperature of warmest quarter	0.92
*Capurodendron greveanum-mandrarense*	22	Annual precipitation	0.97
		Precipitation of wettest quarter	0.02
		Precipitation of wettest month	0.91
*Capurodendron mandrarense*	60	Max temperature of warmest Month	0.88
		Temperature seasonality	0.87
		Temperature annual range	0.82
*Capurodendron microphyllum*	14	Precipitation seasonality	0.94
		Precipitation of wettest month	0.94
		Precipitation of wettest quarter	0.91
*Capurodendron oblongifolium*	6	Precipitation of wettest month	0.99
		Precipitation of wettest quarter	0.97
		Precipitation seasonality	0.96
*Capurodendron perrieri*	45	Precipitation seasonality	0.96
		Mean temperature of warmest quarter	0.94
		Mean temperature of wettest quarter	0.94
*Capurodendron pervillei*	36	Precipitation of wettest month	0.98
		Precipitation of wettest quarter	0.97
		Annual mean temperature	0.97

## Data Availability

All data are indicated in the manuscript or available in the Supplementary Materials.

## Supplementary Materials

The following are available online at https://www.mdpi.com/article/10.3390/plants10081702/s1, Figure S1: Pseudocoalescent phylogenetic tree from Astral inferred from RAxML analyses of A. 600 gene exonic regions, B. 608 gene supercontigs including introns, exons and flanking regions and C. 195 microsatellite flanking regions. All specimens contained less than 20% missing nucleotide positions. Figure S2: STRUCTURE output from k = 2 to k = 6 for the Arid and Western Species Complexes ordered by morphospecies, with specimen numbers given according to Table 2 and indicated at the end of each dataset. The probability of each k according to the ΔK method ([48] Evanno et al., 2005) is given, Table S1: Specimen information used in the morphological PCA. Character number and states are given in Table 2. Table S2: Results for the ABBA BABA tests. Numbers in taxa names correspond to the groups shown in Figure 5. *p*-value threshold for Z-scores after Benjamini and Bonferroni corrections are indicated in bold and bold underlined, respectively.
