# Supplementary material for: Species Delimitation and Conservation in Taxonomically Challenging Lineages: The Case of Two Clades of Capurodendron (Sapotaceae) in Madagascar
Source: Plants (Basel). 2021 Aug 18;10(8):1702. doi: 10.3390/plants10081702 (PMC8400537; doi:10.3390/plants10081702)
Supplement: Supplementary file 1 [file plants-10-01702-s001.zip › TableS1.pdf]

Table S1. Specimen information used in the morphological PCA. Character number and states are given in Table 2.

Specimen	Species	1	2	3	4	5	6	7	8	9	10	11	12	13	14	15	16	17	18	19	20	21	22
**Phillipson 3611**	*C. androyense*	1.5	1	1	2	2	0	1	11	6	2	2	0	0	0	0	1	1	4	4	0	3	3
**SF 11856**	*C. androyense*	NA[1]	1	1	2	1	0	1	9	6	2	2	0	1	0	0	1	1	4	5	0	3	3
**SF 20438**	*C. androyense*	4.5	1	1	2	2	0	1	10	5	2	2	0	1	0	0	1	1	4	NA	NA	NA	NA
**SF11867**	*C. androyense*	NA	1	1	2	1	0	1	10	6	2	2	0	0	0	0	0	1	4	4	0	3	2
**Decary 9587**	*C. androyense*	NA	1	1	2	1	0	0	11	6	2	2	0	0	0	0	0	0	3	3	0	3	2
**SF 11705**	*C. androyense*	NA	1	1	1	2	0	1	6	4	2	2	0	1	0	0	0	1	2	2	0	2	2
**Miller 3781**	*C. androyense*	1.5	1	1	2	2	0	1	10	5	2	2	1	1	0	0	1	1	4	NA	NA	NA	NA
**Ratovoson 1451**	*C. androyense*	5	1	1	2	2	0	1	8	5	2	2	1	1	0	0	1	1	3	2	0	2	2
**Rakotomalaza 1718**	*C. androyense*	3.5	1	1	3	1	0	1	13	6	2	2	0	1	0	0	0	1	4	NA	NA	NA	NA
**SF 22258**	*C. androyense*	4.5	1	1	2	2	0	1	13	4	1	0	1	1	0	0	1	1	4	3	0	3	2
**Gautier 6355**	*C. androyense*	3.5	1	1	1	2	0	1	7	3	2	2	1	1	0	0	1	1	2	1	0	2	2
**Gautier 6353**	*C. androyense*	4.5	1	1	1	2	0	1	8	4	2	2	1	1	0	0	1	1	3	NA	NA	NA	NA
**Gautier 6352**	*C. androyense*	4	1	1	1	2	0	1	6	3	2	2	1	1	0	0	1	1	3	NA	NA	NA	NA
**Randrianaivo 213**	*C. androyense*	2.5	1	1	2	2	0	1	10	5	2	2	1	1	0	0	1	1	6	NA	NA	NA	NA
**Gautier 6343**	*C. androyense*	NA	1	1	2	2	0	1	10	4	2	2	1	1	0	0	1	1	4	NA	NA	NA	NA
**Gautier 6346**	*C. androyense*	NA	1	1	1	2	0	1	4	2	1	2	1	1	0	0	1	1	0	NA	NA	NA	NA
**Gautier 6328**	*C. androyense*	1.5	1	1	3	2	0	1	12	5	2	2	1	1	0	0	1	1	3	6	0	3	2
**Rogers 474**	*C. androyense*	1.5	1	1	2	2	0	1	12	4	2	2	1	1	0	0	1	1	4	2	0	2	2
**Rakotomalaza 1719**	*C. androyense*	2	1	1	3	2	0	1	15	4	2	1	1	1	0	0	1	1	3	NA	NA	NA	NA
**Gautier 6374**	*C. androyense*	2.2	1	1	2	2	0	1	15	6	2	2	0	1	0	0	0	1	4	2	0	2	2
**Gautier 6371**	*C. androyense*	0.1	1	1	1	2	0	1	8	6	2	2	0	1	0	0	0	1	3	3	0	2	2
**Randrianaivo 2954**	*C. androyense*	5	1	1	2	2	0	1	9	5	2	2	1	1	0	0	1	1	3	2	0	2	3
**Gautier 6372**	*C. androyense*	1.7	1	1	4	2	0	1	20	13	1	1	0	0	0	0	0	1	5	5	0	3	3
**Gautier 6370**	*C. androyense*	1.5	1	1	1	2	0	1	13	5	2	2	0	0	0	0	0	1	5	2	0	2	2
**Gautier 6376**	*C. androyense*	3.5	1	1	1	2	0	1	7	4	2	2	1	1	0	0	1	1	3	3	0	3	2
**Gautier 6358**	*C. androyense*	1.7	1	1	1	2	0	1	6	3	2	2	0	1	0	0	0	1	2	3	0	2	2
**Gautier 6361**	*C. androyense*	3	1	1	1	2	0	1	10	5	2	2	0	1	0	0	1	1	3	3	0	2	3

**Gautier 6387**	*C. androyense*	2	1	1	3	2	0	0	19	8	2	2	1	1	0	0	1	1	4	4	0	3	3
**Randrianaivo 2959**	*C. androyense*	6	1	1	2	2	0	1	10	5	2	2	0	1	0	0	0	1	5	1	0	2	2
**SF 22230**	*C. androyense-mandrarense*	1	1	4	2	0	1	20	8	NA	2	2	1	1	0	0	1	1	5	5	0	3	2
**SF 22286**	*C. androyense-mandrarense*	7.5	1	1	6	2	0	1	32	14	2	2	1	1	0	0	1	1	6	6	0	3	3
**Phillipson 5603**	*C. greveanum-mandrarense*	4	1	1	25	2	1	2	35	25	1	2	0	0	1	0	1	1	13	6	0	4	3
**Gautier 6339**	*C. greveanum-mandrarense*	15	1	1	18	2	1	1	45	35	1	2	0	0	1	0	1	1	8	4	0	3	3
**Gautier 6336**	*C. greveanum-mandrarense*	3	1	1	18	1	1	1	45	22	1	2	0	0	1	0	1	1	8	6	0	3	3
**Gautier 6341**	*C. greveanum-mandrarense*	7	1	1	18	1	1	1	40	19	1	1	0	0	1	0	0	0	10	4	0	3	3
**Gautier 6333**	*C. greveanum-mandrarense*	14	1	1	20	1	0	1	50	23	1	2	0	0	1	0	1	1	9	5	0	3	3
**Gautier 6331**	*C. greveanum-mandrarense*	18	1	1	11	1	0	0	45	18	2	2	0	0	1	0	1	1	8	3	0	3	3
**Randrianaivo 1187**	*C. greveanum-mandrarense*	2.5	1	1	25	1	1	1	42	28	1	2	0	0	1	0	1	1	11	NA	NA	NA	NA
**Andrianjafy 1679**	*C. greveanum-mandrarense*	10	1	1	21	2	1	1	40	25	1	1	0	0	1	0	0	1	11	6	0	3	3
**Andrianjafy 1669**	*C. greveanum-mandrarense*	7	1	1	16	2	1	1	40	20	1	1	0	0	1	0	0	1	9	6	0	4	3
**McPherson 17358**	*C. greveanum-mandrarense*	4	1	1	10	2	1	1	22	14	1	2	0	0	1	0	0	1	7	5	0	4	3
**Gautier 6337**	*C. greveanum-mandrarense*	12	1	1	20	1	1	0	45	20	1	1	0	0	1	0	0	1	9	NA	NA	NA	NA
**Razafindraibe 165**	*C. greveanum-mandrarense*	8	1	1	20	1	1	1	45	23	1	1	0	0	1	0	1	1	10	6	1	5	4
**Gautier 6332**	*C. greveanum-mandrarense*	9	1	1	15	1	0	1	35	20	1	2	0	0	1	0	1	1	11	3	0	3	3
**Gautier 6329**	*C. greveanum-mandrarense*	12	1	1	20	1	0	1	43	19	1	1	0	0	1	0	0	0	11	3	0	3	3
**SF 28965**	*C. greveanum-mandrarense*	NA	1	1	14	2	1	0	22	20	1	2	0	0	1	0	1	1	9	3	1	3	3
**Thomas 30885**	*C. greveanum-mandrarense*	2	1	1	18	1	0	1	39	19	1	1	0	0	1	0	1	1	10	6	1	3	2
**Thomas 30799**	*C. greveanum-mandrarense*	2	1	1	18	1	0	0	46	22	1	2	0	0	1	0	1	1	10	4	1	3	2
**SF20831**	*C. greveanum-mandrarense*	9	1	1	28	1	1	1	55	32	1	2	0	0	1	0	1	1	11	6	1	3	3
**Schatz 2477**	*C. greveanum-mandrarense*	5	1	1	22	2	0	1	41	21	1	2	0	0	1	0	1	1	9	6	1	3	3
**42R224**	*C. mandrarense*	NA	1	1	13	2	0	1	45	20	2	1	0	1	1	1	1	1	9	NA	NA	NA	NA
**SF 28954**	*C. mandrarense*	7.5	1	1	16	2	0	1	55	30	1	1	1	1	1	1	1	1	9	NA	NA	NA	NA
**Randrianasolo 204**	*C. mandrarense*	2.5	1	1	11	2	1	1	36	19	1	1	1	1	1	1	1	1	7	3	1	2	2
**Ratovoson 1473**	*C. mandrarense*	4	1	1	10	2	0	1	30	10	1	1	1	1	1	1	1	1	8	3	1	3	3
**Andriamahajarivo 1532**	*C. mandrarense*	7	1	1	7	2	1	1	42	20	1	1	1	1	1	1	1	1	7	3	1	3	3
**Randrianaivo 2956**	*C. mandrarense*	8	1	1	14	1	1	1	50	25	1	1	1	1	1	1	1	1	8	NA	NA	NA	NA
**Randrianaivo 1764**	*C. mandrarense*	4	1	1	16	1	1	1	36	15	1	1	1	1	1	1	1	1	10	NA	NA	NA	NA

**Randrianaivo 1785**	*C. mandrarense*	4.5	1	1	14	1	0	1	29	17	1	1	1	1	1	1	1	1	8	7	1	3	3
**Randrianaivo 2966**	*C. mandrarense*	3	1	1	10	2	0	1	59	30	1	1	0	1	1	1	1	1	7	NA	NA	NA	NA
**Randrianaivo 2964**	*C. mandrarense*	3	1	1	13	2	0	1	30	24	1	1	0	1	1	1	1	1	7	4	1	3	3
**Randrianaivo 2962**	*C. mandrarense*	3.5	1	1	6	2	0	2	25	18	1	2	0	1	1	1	1	1	7	3	1	3	3
**Randrianaivo 2967**	*C. mandrarense*	3	1	1	10	2	0	1	42	19	1	1	0	1	1	1	1	1	9	NA	NA	NA	NA
**Randrianaivo 2958**	*C. mandrarense*	8	1	1	9	1	0	0	43	20	1	1	0	1	1	1	1	1	8	NA	NA	NA	NA
**Randrianaivo 2963**	*C. mandrarense*	2.5	1	1	11	1	0	1	54	29	1	2	0	1	1	1	1	1	9	NA	NA	NA	NA
**Randrianaivo 2965**	*C. mandrarense*	2.5	1	1	6	1	0	1	32	14	1	1	0	1	1	1	1	1	7	NA	NA	NA	NA
**Randrianaivo 2961**	*C. mandrarense*	3.5	1	1	13	1	1	1	45	27	1	1	0	1	1	1	1	1	5	NA	NA	NA	NA
**Randrianaivo 2957**	*C. mandrarense*	9	1	1	13	1	0	1	53	39	1	1	0	1	1	1	1	1	7	NA	NA	NA	NA
**RN5035**	*C. mandrarense*	NA	1	1	12	2	0	1	40	19	1	1	0	1	1	1	1	1	7	5	1	3	3
**Gautier 5793**	*C. mandrarense*	4	0	1	10	2	0	1	44	24	1	1	1	1	1	1	1	1	8	NA	NA	NA	NA
**Rakotomalaza 603**	*C. mandrarense*	4	1	1	11	2	0	1	54	26	1	1	0	1	1	1	1	1	8	2	1	3	3
**Gautier 6356**	*C. mandrarense*	4	1	1	6	2	0	1	25	18	1	1	1	1	1	1	1	1	6	NA	NA	NA	NA
**Randrianaivo 2965**	*C. mandrarense*	2.5	1	1	11	2	0	1	45	27	1	1	0	1	1	1	1	1	8	NA	NA	NA	NA
**Gautier 6378**	*C. mandrarense*	3	0	1	3	2	0	1	25	15	1	1	0	1	1	1	1	1	6	NA	NA	NA	NA
**Andriamihajarivo 498**	*C. mandrarense*	NA	1	1	14	2	0	1	35	18	1	1	1	1	1	1	1	1	6	3	1	3	3
**Rolland 249**	*C. mandrarense*	NA	0	1	9	2	0	1	24	16	1	1	1	1	1	1	1	1	7	NA	NA	NA	NA
**Randrianaivo 899**	*C. mandrarense*	3	1	1	10	2	0	1	27	10	1	1	1	1	1	1	1	1	7	NA	NA	NA	NA
**Dumetz 1414**	*C. mandrarense*	3	1	1	14	2	0	1	39	21	1	1	1	1	1	1	1	1	9	NA	NA	NA	NA
**Rabevohitra 2392**	*C. mandrarense*	5	1	1	14	2	0	1	30	20	1	1	1	1	1	1	1	1	7	2	1	2	2
**Phillipson 2843**	*C. mandrarense*	10	1	1	142	2	0	1	36	16	1	1	1	1	1	1	1	1	7	10	1	3	3
**Gautier 6351**	*C. mandrarense*	6	1	1	10	2	0	1	39	19	1	1	0	1	1	1	1	1	6	NA	NA	NA	NA
**Gautier 6350**	*C. mandrarense*	1	1	1	6	2	0	1	21	11	1	1	1	1	1	1	1	1	5	NA	NA	NA	NA
**Gautier 6349**	*C. mandrarense*	8	1	1	13	2	0	1	42	29	1	1	1	1	1	1	1	1	8	NA	NA	NA	NA
**Randrianaivo 2978**	*C. mandrarense*	10	1	1	12	2	0	1	46	21	1	1	0	1	1	1	1	1	7	NA	NA	NA	NA
**Randrianaivo 2971**	*C. mandrarense*	12	1	1	12	2	0	1	25	20	1	2	1	1	1	1	1	1	7	NA	NA	NA	NA
**Randrianaivo 2980**	*C. mandrarense*	5	1	1	8	2	0	1	32	15	1	1	1	1	1	1	1	1	8	NA	NA	NA	NA
**Randrianaivo 2970**	*C. mandrarense*	15	1	1	10	2	0	2	37	20	1	2	1	1	1	1	1	1	8	NA	NA	NA	NA
**Randrianaivo 2981**	*C. mandrarense*	15	1	1	12	2	0	2	39	25	1	1	1	1	1	1	1	1	7	4	1	3	3

**SF 6692**	*C. mandrarense*	NA	NA	1	5	2	0	2	35	22	1	1	1	1	1	1	1	1	7	NA	NA	NA	NA
**SF 3462**	*C. greveanum*	NA	0	0	32	0	1	2	115	70	1	1	0	0	1	0	0	0	10	NA	NA	NA	NA
**Ralimanana 70**	*C. greveanum*	10	0	0	35	0	1	1	59	41	1	1	0	0	1	0	0	0	10	9	0	3	2
**Razafimandimbison 2277**	*C. greveanum*	6.5	0	0	34	0	1	1	52	39	1	1	0	0	0	0	0	0	10	5	0	3	3
**Ratovoson 2115**	*C. greveanum*	1.5	0	0	41	0	1	1	55	36	1	1	0	0	1	0	0	0	14	NA	NA	NA	NA
**Razafitsalama 854**	*C. greveanum*	3	0	0	35	0	0	2	64	40	1	1	0	0	1	0	0	0	14	NA	NA	NA	NA
**Callmander 471**	*C. greveanum*	4	0	0	37	0	1	2	67	35	1	1	0	0	1	0	0	0	13	4	0	3	3
**Razafitsalama 595**	*C. greveanum*	3	0	0	30	0	1	2	55	38	1	1	0	0	1	0	0	0	15	6	0	3	2
**Schatz 4200**	*C. greveanum*	2.5	0	0	38	0	0	2	68	42	1	1	0	0	1	0	0	0	13	9	0	3	2
**Rakotonandrasana 1203**	*C. greveanum*	4	0	0	51	0	1	1	54	33	1	1	0	0	1	0	0	0	11	NA	NA	NA	NA
**Jullet 255**	*C. greveanum*	4	0	0	23	0	0	2	49	35	1	2	0	0	1	0	0	0	12	NA	NA	NA	NA
**Gautier 5485**	*C. greveanum*	3	0	0	30	0	1	2	61	37	1	1	0	0	1	0	0	0	13	NA	NA	NA	NA
**SF 13123**	*C. greveanum*	NA	0	0	31	0	1	2	74	44	1	1	0	0	1	0	0	0	13	8	0	3	3
**Randrianaivo 2977**	*C. greveanum*	15	0	0	36	0	0	1	77	41	1	1	0	0	1	0	0	0	10	9	0	3	3
**Applequist 158**	*C. greveanum*	2	0	0	35	0	0	1	67	30	1	1	0	0	1	0	0	0	17	NA	NA	NA	NA
**Ratovoson 832**	*C. greveanum*	2	0	0	22	0	0	1	57	31	1	1	0	0	1	0	0	0	16	NA	NA	NA	NA
**SF 5878**	*C. greveanum*	NA	0	0	25	0	1	1	62	29	1	1	0	0	1	0	0	0	14	NA	NA	NA	NA
**Randrianaivo 476**	*C. greveanum*	15	0	0	14	0	1	0	46	29	1	2	0	0	1	0	0	0	15	NA	NA	NA	NA
**Randrianaivo 2974**	*C. greveanum*	12	0	0	34	0	1	1	76	33	1	1	0	0	1	0	0	0	13	7	0	3	2
**Ranaivojaon n?**	*C. greveanum*	7	0	0	27	0	1	2	59	25	1	1	0	0	1	0	0	0	9	8	0	3	2
**Randranaivo 2982**	*C. greveanum*	10	0	0	20	0	0	1	56	31	1	1	0	0	1	0	0	0	12	NA	NA	NA	NA
**Jongkind 3623**	*C. greveanum*	4.5	0	0	36	0	1	1	56	33	1	1	0	0	1	0	0	0	14	NA	NA	NA	NA
**Randrianaivo 2975**	*C. greveanum*	25	0	0	29	0	1	1	51	36	1	1	0	0	1	0	0	0	11	NA	NA	NA	NA
**Rabevohitra 4519**	*C. greveanum*	NA	0	0	36	0	1	2	64	50	1	1	0	0	1	0	0	0	14	7	0	3	2
**Randrianaivo 600**	*C. greveanum*	2	0	0	24	0	1	1	70	30	1	2	0	0	1	0	0	0	15	NA	NA	NA	NA
**Cheek 1487**	*C. greveanum*	4	0	0	47	0	0	0	70	39	1	2	0	0	1	0	0	0	20	NA	NA	NA	NA
**SF 12796**	*C. greveanum*	NA	0	0	34	0	0	1	61	35	1	1	0	0	1	0	0	0	14	6	0	3	2
**SF 6900**	*C. greveanum*	NA	0	0	42	0	0	1	76	35	1	1	0	0	1	0	0	0	11	7	0	3	2
**Rabenontoandro 610**	*C. greveanum*	0.5	0	0	26	0	1	1	50	20	1	1	0	0	1	0	0	0	12	NA	NA	NA	NA
**Gautier 6382**	*C. microphyllum*	3	0	1	3	1	0	1	16	3	1	0	0	0	0	0	0	0	2	4	0	2	2

**Randrianaivo 890**	*C. microphyllum*	3	0	1	3	1	0	1	17	5	1	1	0	0	1	0	0	0	3	NA	NA	NA	NA
**Gautier 6393**	*C. microphyllum*	1.8	0	1	4	2	0	1	17	6	1	1	0	1	0	0	1	0	3	NA	NA	NA	NA
**Gautier 5794**	*C. microphyllum*	1.8	0	1	2	1	0	1	15	7	1	0	0	0	1	0	0	0	4	NA	NA	NA	NA
**Gautier 6381**	*C. microphyllum*	2.5	0	1	2	1	0	1	20	5	1	0	0	0	0	0	0	0	3	3	0	2	2
**Gautier 6383**	*C. microphyllum*	2.5	0	1	2	1	0	1	20	5	1	0	0	0	1	0	0	0	3	5	0	2	2
**Thulin 11814**	*C. microphyllum*	3	0	1	2	2	0	1	14	4	1	0	0	0	0	0	0	0	3	3	0	2	2

[1] Non avalable data.
