# Supplementary material for: Species Delimitation and Conservation in Taxonomically Challenging Lineages: The Case of Two Clades of Capurodendron (Sapotaceae) in Madagascar
Source: Plants (Basel). 2021 Aug 18;10(8):1702. doi: 10.3390/plants10081702 (PMC8400537; doi:10.3390/plants10081702)
Supplement: Supplementary file 1 [file plants-10-01702-s001.zip › TableS2.pdf]

Table S2. Results for the ABBA BABA tests. Numbers in taxa names correspond to the groups shown in Fig. 5. P-value threshold for Z-scores after Benjamini and Bonferroni corrections are indicated in bold and bold underlined, respectively.

P1	P2	P3	D-statistics	Z-score	p-value	f4-ratio	BBAA	ABBA	BABA
C. mandrarense 1	*C. mandrarense 2*	*C. androyense 1*	0.0497606	6.42715	**<u>6.50E-11</u>**	0.251148	788.581	760.915	688.778
C. greveanum-mandrarense 2	*C. androyense 2*	*C. microphyllum*	0.10012	6.18189	**<u>3.17E-10</u>**	0.0685898	1251.15	640.625	524.02
C. greveanum-mandrarense 2	*C. androyense 1*	*C. microphyllum*	0.121506	4.97969	**<u>3.18E-07</u>**	0.0865021	1189.5	680.445	533.004
C. mandrarense 1	*C. androyense 1*	*C. microphyllum*	0.0763872	4.10704	**<u>2.00E-05</u>**	0.0558021	1182.49	634.857	544.75
C. greveanum-mandrarense 2	*C. mandrarense 2*	*C. microphyllum*	0.0776264	3.55601	**<u>0.00018827</u>**	0.0528676	1255.61	628.711	538.134
C. mandrarense 1	*C. androyense 2*	*C. microphyllum*	0.0510879	3.5151	**<u>0.00021979</u>**	0.0363943	1221.69	609.713	550.443
C. greveanum-mandrarense 1	*C. androyense 2*	*C. microphyllum*	0.0812243	3.36528	**<u>0.00038233</u>**	0.0558256	1270.52	625.496	531.518
C. greveanum-mandrarense 1	*C. androyense 1*	*C. microphyllum*	0.103722	3.24326	**<u>0.00059085</u>**	0.0759686	1209.03	664.464	539.578
C. mandrarense 2	*C. androyense 1*	*C. microphyllum*	0.0492489	3.06844	**0.00107591**	0.0346187	1225.69	605.738	548.875
C. androyense 1	*C. androyense 2*	*C. greveanum-mandrarense 1*	0.0477013	3.02814	**0.00123032**	0.0383211	803.191	763.079	693.594
C. mandrarense 2	*C. mandrarense 1*	*C. greveanum-mandrarense 2*	0.0399563	2.97038	**0.00148717**	0.107273	789.355	770.527	711.318
C. androyense 1	*C. androyense 2*	*C. greveanum-mandrarense 2*	0.0493885	2.91606	**0.0017724**	0.115775	824.512	749.663	679.099
C. mandrarense 1	*C. mandrarense 2*	*C. androyense 2*	0.0234767	2.74341	**0.00304024**	0.489905	781.107	749.087	714.722
C. androyense 1	*C. mandrarense 2*	*C. greveanum-mandrarense 2*	0.0415562	2.71572	**0.00330662**	0.0992773	810.102	759.73	699.107
C. mandrarense 1	*C. mandrarense 2*	*C. microphyllum*	0.0295901	2.60751	**0.00456021**	0.0203313	1283.16	578.354	545.11
C. greveanum-mandrarense 2	*C. mandrarense 1*	*C. microphyllum*	0.0506092	2.1527	0.015671	0.0340983	1314.06	595.111	537.776
C. mandrarense 2	*C. androyense 2*	*C. greveanum*	0.0398239	2.10546	0.0176257	0.011273	1864.51	460.892	425.589
C. androyense 1	*C. mandrarense 2*	*C. greveanum-mandrarense 1*	0.0335353	2.08526	0.0185228	0.027944	788.711	763.158	713.633
C. greveanum-mandrarense 1	*C. mandrarense 2*	*C. microphyllum*	0.058588	2.01479	0.0219631	0.0406663	1264.83	613.383	545.487
C. mandrarense 1	*C. androyense 2*	*C. greveanum*	0.0437652	1.9997	0.0227661	0.0123701	1835.66	466.289	427.186
C. mandrarense 2	*C. androyense 2*	*C. microphyllum*	0.0226033	1.9531	0.0254041	0.0160039	1235.13	588.737	562.711
C. greveanum-mandrarense 1	*C. mandrarense 1*	*C. mandrarense 2*	0.0358089	1.65592	0.0488694	0.464718	795.071	766.329	713.344
C. mandrarense 2	*C. androyense 1*	*C. greveanum*	0.0441139	1.65297	0.049169	0.0125995	1869.97	466.065	426.683
C. greveanum-mandrarense 2	*C. greveanum-mandrarense 1*	*C. mandrarense 1*	0.0391536	1.6399	0.0505129	0.23806	1133.58	635.279	587.406
C. mandrarense 1	*C. androyense 1*	*C. greveanum*	0.0474895	1.62469	0.0521142	0.0138184	1808.08	476.246	433.063
C. greveanum-mandrarense 2	*C. greveanum-mandrarense 1*	*C. androyense 2*	0.0300688	1.54731	0.0608945	0.470152	1162.01	601.947	566.804
C. greveanum-mandrarense 2	*C. greveanum-mandrarense 1*	*C. androyense 1*	0.0315978	1.52726	0.0633487	0.111474	1218.46	588.905	552.829
C. androyense 2	*C. androyense 1*	*C. microphyllum*	0.0268765	1.38969	0.0823115	0.0192112	1243.03	589.097	558.26
C. greveanum-mandrarense 2	*C. mandrarense 1*	*C. androyense 1*	0.0268571	1.30396	0.0961233	0.120374	823.823	742.849	703.991
C. greveanum-mandrarense 2	*C. greveanum-mandrarense 1*	*C. mandrarense 2*	0.021273	1.29567	0.0975444	0.180843	1185.35	605.318	580.1
C. androyense 1	*C. androyense 2*	*C. mandrarense 1*	0.0232678	1.26445	0.103035	0.114489	807.953	734.815	701.398

C. mandrarense 1	*C. greveanum-mandrarense 2*	*C. greveanum*	0.0420314	1.18379	0.118248	0.0117706	1878.51	459.428	422.365
C. mandrarense 1	*C. greveanum-mandrarense 1*	*C. greveanum*	0.0445085	1.15065	0.124939	0.0118998	1905.82	439.288	401.85
C. mandrarense 2	*C. greveanum-mandrarense 2*	*C. greveanum*	0.0367866	1.11374	0.132695	0.0105586	1828.5	468.74	435.476
C. mandrarense 2	*C. greveanum-mandrarense 1*	*C. greveanum*	0.0384823	0.995624	0.159716	0.0106487	1836.01	451.44	417.982
C. greveanum-mandrarense 1	*C. mandrarense 1*	*C. microphyllum*	0.0307927	0.951731	0.170617	0.0204725	1342.69	576.441	542.001
C. greveanum-mandrarense 2	*C. greveanum-mandrarense 1*	*C. microphyllum*	0.0251229	0.945938	0.17209	0.0134342	1764.43	465.984	443.144
C. mandrarense 1	*C. greveanum-mandrarense 1*	*C. androyense 2*	0.0227826	0.884659	0.18817	0.470842	808.396	746.629	713.366
C. microphyllum	*C. androyense 1*	*C. greveanum*	0.0288724	0.762963	0.222743	0.0100605	1336.31	562.276	530.719
C. mandrarense 2	*C. androyense 2*	*C. greveanum-mandrarense 1*	0.0132666	0.75393	0.225446	0.0114424	763.428	762.264	742.303
C. microphyllum	*C. androyense 2*	*C. greveanum*	0.0247916	0.584897	0.279308	0.00866873	1314.29	566.972	539.539
C. microphyllum	*C. greveanum-mandrarense 2*	*C. greveanum*	0.022216	0.474558	0.317551	0.00817318	1221.73	589.019	563.417
C. microphyllum	*C. greveanum-mandrarense 1*	*C. greveanum*	0.0233908	0.471508	0.318639	0.00842537	1232.25	577.128	550.747
C. mandrarense 2	*C. androyense 2*	*C. greveanum-mandrarense 2*	0.00672089	0.442292	0.329139	0.0180755	780.591	744.514	734.573
C. mandrarense 1	*C. mandrarense 2*	*C. greveanum*	0.00439563	0.292312	0.385024	0.00119624	1905.26	434.145	430.345
C. mandrarense 1	*C. microphyllum*	*C. greveanum*	0.0109345	0.269344	0.393832	0.00386178	1277.83	562.215	550.053
C. androyense 2	*C. androyense 1*	*C. greveanum*	0.00466415	0.203858	0.419232	0.00132371	1877.49	439.366	435.287
C. androyense 2	*C. androyense 1*	*C. mandrarense 2*	0.00296491	0.174235	0.43084	0.045986	770.949	736.531	732.176
C. greveanum-mandrarense 2	*C. androyense 1*	*C. greveanum*	0.00647664	0.170526	0.432298	0.00196649	1768.43	475.492	469.372
C. mandrarense 2	*C. microphyllum*	*C. greveanum*	0.0068663	0.170471	0.43232	0.00242761	1305.27	556.72	549.127
C. greveanum-mandrarense 1	*C. androyense 1*	*C. greveanum*	0.00670677	0.148989	0.440781	0.00196864	1791.73	462.877	456.71
C. greveanum-mandrarense 2	*C. androyense 2*	*C. greveanum*	0.00227163	0.082643	0.467068	0.00066153	1817.59	450.044	448.004
C. greveanum-mandrarense 1	*C. mandrarense 1*	*C. androyense 1*	0.00203414	0.0804979	0.467921	0.0105181	842.496	714.143	711.244
C. greveanum-mandrarense 1	*C. androyense 2*	*C. greveanum*	0.00205751	0.0621516	0.475221	0.00057633	1839.27	436.329	434.537
C. greveanum-mandrarense 2	*C. mandrarense 1*	*C. androyense 2*	0.00115809	0.0578715	0.476925	0.0233336	787.145	739.588	737.877
C. greveanum-mandrarense 2	*C. greveanum-mandrarense 1*	*C. greveanum*	0.00108867	0.0341529	0.486378	0.00024021	2307.51	342.871	342.125

A significant D > 0 is a sign of introgression between populations (P). D measures an excess of allele sharing of P3 with P1 and P2. Z measures how many standard deviations D is from its expectation, which is 0. The f4 is an estimation of the proportion of admixture. The last three columns represent the different combinations of mutations tested.
