# Supplementary figures and images for: Species Delimitation and Conservation in Taxonomically Challenging Lineages: The Case of Two Clades of Capurodendron (Sapotaceae) in Madagascar
Source: Plants (Basel). 2021 Aug 18;10(8):1702. doi: 10.3390/plants10081702 (PMC8400537; doi:10.3390/plants10081702)

## Capurodendron Arid Complex

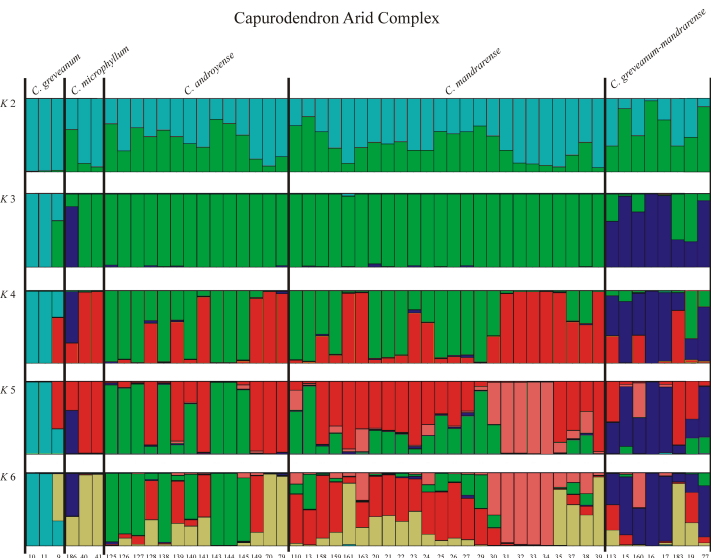

## Capurodendron Western Complex

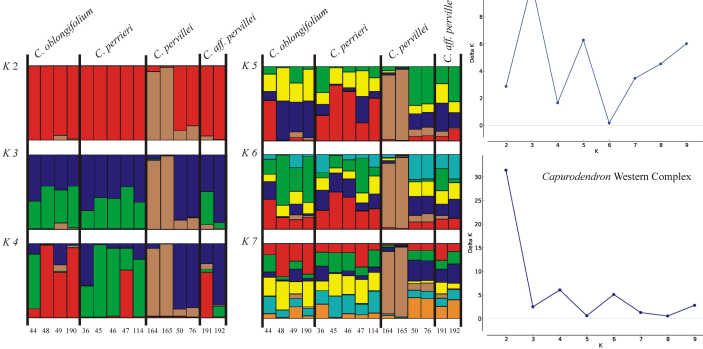

Supplement: Supplementary file 1 [file plants-10-01702-s001.zip › FigS2.pdf]
